# Antimicrobial Resistance in Selected *Enterobacteriaceae* from Broilers and Their Environment: ESBL, AmpC, Carbapenemases, Colistin, and Fluoroquinolone Resistance—A Systematic Review and Meta-Analysis

**DOI:** 10.3390/antibiotics14121268

**Published:** 2025-12-15

**Authors:** Julia von Kiparski, Nunzio Sarnino, Diana Vargas, Aleksandra Atanasova, Roswitha Merle

**Affiliations:** 1 Institute of Veterinary Epidemiology and Biostatistics, Veterinary Centre for Resistance Research, School of Veterinary Medicine, Freie Universität Berlin, 14163 Berlin, Germany; nunzio.sarnino@fu-berlin.de; 2 Institute for Animal Hygiene and Environmental Health, Veterinary Centre for Resistance Research, School of Veterinary Medicine, Freie Universität Berlin, 14163 Berlin, Germany; diana.vargas.gallo@fu-berlin.de; 3 Leibniz Institute for Agricultural Engineering and Bioeconomy (ATB), 14469 Potsdam, Germany; aatanasova@atb-potsdam.de

**Keywords:** AMR, broiler, *Enterobacteriaceae*, beta-lactams, colistin, fluoroquinolone

## Abstract

**Background/Objectives:** Antimicrobial resistance (AMR) threatens global public health. This systematic review and meta-analysis, as part of the “ENVIRE” project (interventions to control the dynamics of antimicrobial resistance from chickens through the environment), assesses the prevalence of phenotypic and genotypic resistance, including extended-spectrum beta-lactamases (ESBLs), AmpC beta-lactamases, carbapenemases, colistin, and fluoroquinolone resistance, in broiler chickens and their environment. **Methods:** The analysis covers the years 2002–2022, focusing on *Escherichia* (*E.*) *coli*, *Klebsiella* spp., *Enterobacter* spp., and *Citrobacter* spp. in fecal, meat, environmental, and other-than-feces samples from observational studies published in PubMed and Web of Science. Quality assessment was performed using the Alberta Heritage Foundation criteria. **Results:** Data from 170 studies, conducted in Europe, North Africa, and North America, were included. The most frequently studied resistance was to beta-lactam, with focus on ESBL-producing and AmpC beta-lactamase isolates. The pooled prevalence of ESBL-resistant *E. coli* observed in meat samples at 41% and in fecal samples at 38% demonstrated significant heterogeneity between the studies. The negative binomial regression analysis of prevalence data revealed significantly higher ESBL-producing *E. coli* rates in European meat samples compared to North African samples. **Conclusions:** This systematic review revealed substantial variation in prevalence and emphasizes the need for standardized surveillance systems and robust study designs.

## 1. Introduction

The global rise of antimicrobial resistance (AMR) has become one of the most pressing challenges in public health, threatening the effective treatment of infectious diseases in both human and veterinary medicine [1,2]. The prevalence of antimicrobial resistance in broiler production is a significant concern for public health, as recent studies on commercial broilers have shown that multidrug-resistant *E. coli* act as potential reservoirs along the food chain and pose a risk of transmission to humans [3,4]. A recent review from China has also described diverse antimicrobial determinants in *E. coli* from poultry production, further underlining the global relevance of AMR in poultry and other food-producing animals [5]. Among extended-spectrum beta-lactamase (ESBL), CTX-M variants are the most detected ones in humans and poultry [6,7]. In livestock, and particularly in poultry production, antibiotics are widely used to prevent and treat infections, particularly in intensive farming systems where large numbers of broilers are raised under confined conditions [8,9]. Chicken meat represents an affordable and globally consumed source of animal protein, driving the expansion of intensive production, which often relies heavily on antibiotics to maintain flock health [10].

This widespread use of antibiotics has raised concerns that broilers could serve as reservoirs of antimicrobial-resistant bacteria, potentially transmitting resistance to humans through food, direct contact, or environmental pathways [11]. Moreover, *E. coli* has been described as particularly capable of persisting in hosts and the environment due to virulence factors and biofilm formation, which promote adhesion and reduced susceptibility to antimicrobial treatment [12]. Despite the growing body of literature on AMR, the research landscape remains fragmented. Existing studies often have limited geographical coverage, inconsistent methodologies, and a lack of epidemiological focus, hindering the accurate assessment of resistance prevalence and comparability across regions [13]. These gaps hinder reliable prevalence estimates and complicate cross-study comparisons. Furthermore, low- and middle-income countries often face gaps in surveillance and regulatory frameworks, leaving critical resistance data underrepresented [14]. A systematic approach is needed to address these gaps and identify methodological challenges [13]. The project “ENVIRE” (interventions to control the dynamics of antimicrobial resistance from chickens through the environment), part of the European Transnational Programme JPIAMR-ACTION (Joint Programming Initiative on Antimicrobial Resistance, https://www.jpiamr.eu/), focuses on interventions to minimize the selection and dissemination of antimicrobial-resistant bacteria in broiler chickens and their surroundings, ultimately addressing risks to human health [15].

The systematic review and meta-analysis presented here, conducted as part of the ENVIRE project, aims to quantify and summarize the reported occurrence of phenotypic resistance patterns and resistance genes in *Enterobacteriaceae*, specifically *Escherichia* (*E.*) *coli*, *Klebsiella* spp., *Enterobacter* spp., and *Citrobacter* spp. (KEC), over the past two decades. It focuses on resistance mechanisms such as beta-lactam resistance mechanisms, including ESBLs and AmpC (Ampicillinase C) beta-lactamases. Colistin resistance, mediated by *mcr-1* (mobilized colistin resistance gene 1), and fluoroquinolone resistance, including plasmid-mediated quinolone resistance (PMQR), are also addressed. Additionally, this study examines carbapenem resistance. Data were collected from broiler chickens, covering various sample types during the fattening period. By synthesizing data from feces and meat samples across multiple regions through a meta-analysis, this approach provides a quantitative assessment of resistance patterns and highlights critical knowledge gaps. Ultimately, this review contributes to a better understanding of the global dynamics of antimicrobial resistance.

## 2. Results

**Figure 1:**
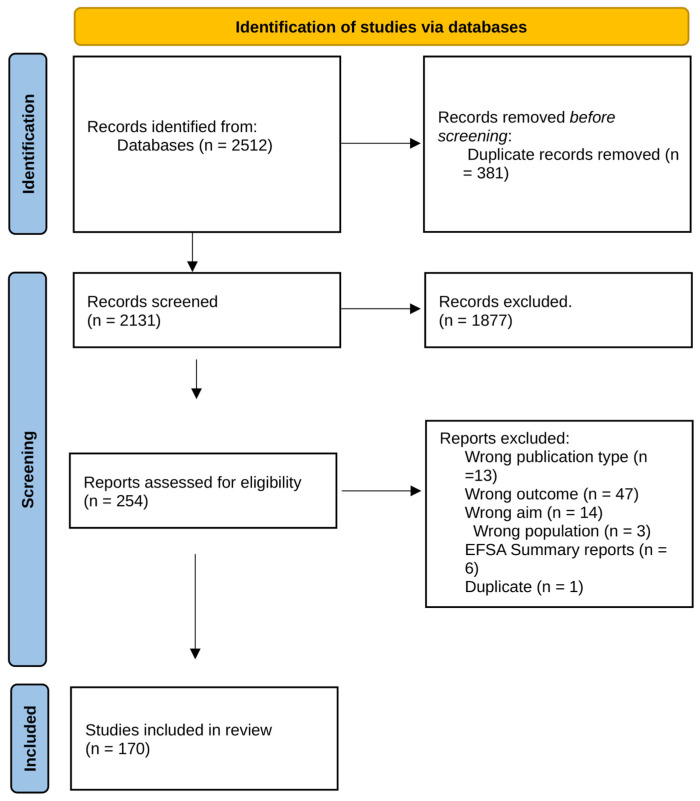
PRISMA flow diagram for the selection process of eligible publications included in this study.

The systematic literature search revealed 2512 publications; 381 were duplicates and removed. Overall, 2131 were screened for eligibility during the title–abstract screening, and 1961 publications were excluded because they did not meet all of the inclusion criteria (e.g., obtained from hatcheries, parent stock, breeding stock, wrong population, wrong study type, geographical limit). Finally, 170 publications (6.7%) were considered for the systematic review (Figure 1).

Regarding publication date, 87% (n = 148/170) of the included publications were from 2012 to July 2022, and 12.9% (n = 22/170) were from 2002 to 2011. Overall, 66.4% (n = 113/170) of the studies were distributed over 25 European countries, followed by North African studies, amounting to 21.8% (n = 37/170), with 19 from Egypt, 9 from Algeria, and 9 from Tunisia. Lastly, 11.8% (n = 20/170) of the studies originated from North America (Canada, USA).

### 2.1. Quality Assessment

**Figure 2:**
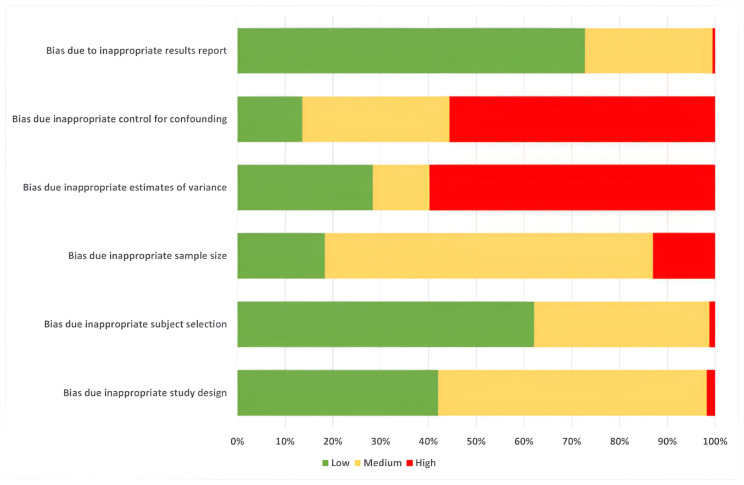
Distribution of scores of the most important criteria for the quality assessment in a systematic review on the occurrence of antimicrobial resistance in selected Enterobacteriaceae in broilers and their environment, including 170 publications.

Overall, 85 publications were regarded as high quality, 55 were acceptable (n = 55), and 30 publications were of critical quality (minimum score 2). Their data are described in this review, but they were excluded from any meta-analysis. Figure 2 shows the distribution of scores of the most essential criteria for the quality assessment. Concerning the sample sizes, we recorded 22 articles with a score of 0 (“obviously inadequate”), 117 articles in which the “samples seemed small or there was no mention of power/sample size/effect size of interest”, and 31 articles in which the sample size seemed appropriate. Only 48 publications provided appropriate variance estimates. A sufficient control for confounding took place in 22 studies.

### 2.2. Beta-Lactam Resistance (ESBL- and AmpC-Producing Enterobacteriaceae)

Most studies reported on beta-lactam resistance (147 articles). A total of 100 publications were published from 2014 to 2022, but only 47 studies were published from 2004 to 2013. Samples from 99 studies were collected in European countries, 28 in North African countries, and 20 in North American countries.

#### 2.2.1. *Enterobacteriaceae*—Prevalence in Feces

We identified 20 studies for a prevalence calculation that investigated fecal samples. Except for four studies—three from North Africa and one from Canada—all the studies originated from Europe. Overall, fecal samples predominantly showed high ESBL/AmpC prevalence in the European studies, with more variable results in North Africa.

Eight studies from Europe and one from North Africa reported high percentages of phenotypic resistant ESBL-*Enterobacteriaceae* (50–100%) in broiler production (Table 1). A subsequent study by Mesa et al. [16] reported the detection of various genetic variants, including 25.56% ESBL genes and 6.77% AmpC genes (Blanc et al. [17]). Five European studies reported moderate percentages of resistant bacteria (between 20% and 50%) among broiler isolates (Table 2). Another five studies reported a relatively low prevalence of resistance, all below 20% (Table 3).

#### 2.2.2. *Enterobacteriaceae*—Prevalence in Slaughterhouses

A total of fifteen studies—two from North Africa (Algeria and Tunisia), two from Canada, and eleven from Europe—investigated samples other than feces (cecal, intestinal, or neck skin samples collected at slaughter). The prevalence data from 13 of these studies are presented in Table 4. Internal slaughterhouse samples (cecal/intestine) from Europe covered the full range from low to high ESBL/AmpC prevalences, but high prevalence categories (50–100%) were more frequently reported than in North Africa or Canada, where studies more often fell into low (<20%) or moderate ranges (20–50%), with only occasionally high-prevalence findings. The two studies that investigated neck skin or carcasses as external slaughterhouse samples showed a high prevalence in Germany on neck skin, in contrast to a moderate range in Canada [36,37]. Two studies could not be included in the quantitative analysis but still provided relevant trends: in their trend analysis from 2011 to 2014, Hanon et al. [38] reported a significant decreasing trend of cefotaxime resistance in 1132 chicken cecal samples in Belgium. Pacholewicz et al. [39] investigated 17 batches in two slaughterhouses in The Netherlands and reported a significant reduction in ESBL *E. coli* along the slaughter process.

#### 2.2.3. *Enterobacteriaceae*—Prevalence in Meat

We summarized 24 studies concerning beta-lactam resistance in meat samples. Four studies each originated from North America and North Africa. The remaining 17 studies belonged to Europe.

High prevalence ranges were mostly observed in southern, central, and northwestern Europe, as well as in several North American studies, whereas moderate and low prevalences were more sporadically reported from all three regions. In detail, thirteen studies reported the prevalence of beta-lactam resistance between 50% and 100%, with ten of these from Europe, three from North America, and one from North Africa (Table 5). Notably, Mollenkopf et al. [40] reported 77.8% AmpC and only 11.1% ESBL phenotypes in *E. coli* from retail meat in the United States, highlighting a clear predominance of AmpC. Seven studies reported moderate levels of resistant *Enterobacteriaceae* between 20% and 50% (Table 6), while four studies reported lower prevalences of resistance (<20%; Table 7).

#### 2.2.4. Clinical Samples

Across the ten studies that investigated isolates from diseased or clinically affected chicken broilers, resistance prevalences were highest in North Africa, while the studies from Europe and North America generally showed much lower levels, mostly below 20%. Notably, three studies from Egypt showed a higher prevalence of resistance, namely, 55% by Amer et al. [41], over 59% by Awad et al. [42], and 76.8% by Badr et al. [43], when analyzing strains from diseased animals with colibacillosis. Dhaouadi et al. [44] reported 30% resistant *Enterobacteriaceae* in Tunisia, while the remaining studies showed lower resistances (below 20%). Another study from Egypt demonstrated phenotypic ESBL and AmpC resistance in 18% of isolates [45]. In Canada, Varga et al. [46] found that 15% of isolates from chickens with suspected colibacillosis were resistant to ceftiofur. In the United States, Huang et al. [47] reported a ceftiofur resistance rate of 4.8%. In Germany, resistances ranged from 0.8% [48] to 4.9% [49]. Additionally, Niero et al. [50] reported genotypic evidence of ESBL/AmpC genes in 9% of broiler samples in Italy.

**Table 4:** ESBL and AmpC resistance in slaughterhouse samples (13 studies).

Author	DOI	Year	Country	Sample Type	Bacteria	n Samples (Isolates)	% Pheno-ESBL	% Pheno-AmpC	% Geno-ESBL	% Geno-AmpC	Denominator
Maciuca et al. [51]	10.1089/mdr.2014.0248	2015	Romania	cecal	*E. coli*	127 (90)	70.87%	37.79%	70.87%	37.79%	S
Reich et al. [36]	10.1016/j.fm.2015.10.020	2015	Germany	neck skin/cecal	*E. coli*	1800	neck skin: 93.30%, cecal 90.00%				S
Alba et al. [52]	10.3389/fmicb.2018.01217	2018	Italy	cecal	*E. coli*	300 (300)	81.33%				S, I
Messaili et al. [53]	10.12834/VetIt.799.3865.2	2019	Algeria	intestine	*E. coli*	100 (100)	73.00%		70.00%		S, I
Boulianne et al. [54]	n.a.	2015	Canada	cecal	*E. coli*	1500 (962)	45.11%				I
Romero-Barrios et al. [37]	10.1089/fpd.2019.2776	2020	Canada	carcasses	*E. coli*	(1135)	35.95%				I
Belmahdi et al. [55]	10.1016/j.jgar.2016.04.006	2016	Algeria	cecal	*E. coli*	61 (61)	32.79%		80.00%	20.00%	I
Parker et al. [56]	10.1136/vr.103579	2016	UK	cecal	*E. coli*	125	28.80%	9.60%	28.00%	6.40%	S
Thorsteinsdottir et al. [57]	10.1111/j.1863-2378.2009.01256.x	2008	Iceland	cecal	*E. coli*	146 (185)	0.00%				S
Randall et al. [58]	10.1093/jac/dkq396	2010	UK	cecal	*E. coli*	388	4.38%		3.61%		S
Dierikx et al. [59]	10.1016/j.vetmic.2010.03.019	2010	The Netherlands	cecal	*E. coli*	(153)	14.38%		95.45%	22.73%	I
Girlich et al. [60]	10.1128/AEM.02491-06	2007	France	cecal	*E. coli*	112	10.71%		10.71%		S
Päivärinta et al. [61]	10.1016/j.ijfoodmicro.2019.108361	2019	Finland	cecal	*E. coli*	605 (605)	18.00%		0.83%	18.84%	S

Abbreviations: Pheno-ESBL/AmpC = phenotypic ESBL or AmpC resistance; Geno-ESBL/AmpC = genotypic ESBL or AmpC detection; Year = year of publication; Country = country where samples were collected; n.a. = no DOI available; Denominator: S = sample-based prevalence (number of resistances per total number of samples); I = isolate-based prevalence (number of resistances per total number of isolates).

**Table 5:** ESBL and AmpC resistance in meat samples (50–100%, 13 studies).

Author	DOI	Year	Country	Bacteria	n Samples (Isolates)	% Pheno-ESBL	% Pheno-AmpC	% Geno-ESBL	% Geno-AmpC	Denominator
Egea et al. [62]	10.1016/j.ijfoodmicro.2012.08.002	2012	Spain	*E. coli*	15	93.33%				S
Ghodousi et al. [63]	10.1089/fpd.2015.1936	2015	Italy	*E. coli*	163 (134)	91.79%		98.51%	11.19%	I
Casella et al. [64]	10.1016/j.ijfoodmicro.2017.07.005	2017	France	*E. coli*	48 (77)	91.67%	4.05%	100%	3.90%	I
Clemente et al. [65]	10.3390/antibiotics10111333	2021	Portugal	*E. coli*	198 (60)	76.67%	21.67%	100%	1.67%	I
Kaesbohrer et al. [66]	10.1016/j.vetmic.2019.03.025	2019	Germany	*E. coli*	199 (192)	74.9%		71.87%	26.04%	S
Belmar Campos et al. [67]	10.1016/j.ijmm.2014.04.012	2014	Germany	*E. coli*, *Klebsiella* spp.	120 (87)	*E. coli* 60.00%, *Klebsiella* spp. 1.67%		100% ^a^		S
Overdevest et al. [68]	10.3201/eid1707.110209	2011	The Netherlands	*E. coli*, *Klebsiella* spp.	89 (71)	*E. coli* 76.40%, *Klebsiella* spp. 6.74%		83.15% ^a^		S
Vogt et al. [69]	10.1089/mdr.2013.0210	2014	Switzerland	*E. coli*	75 (68)	73.33%		73.33%	17.33%	S
Randall et al. [70]	10.1016/j.ijfoodmicro.2016.10.036	2016	UK	*E. coli*	159	65.41%		65.41%		S
Zarfel et al. [71]	10.3390/ijerph111212582	2014	Austria	*E. coli*	50	52.00%		52.00%		S
Forward et al. [72]	10.1155/2004/695305	2004	Canada	*E. coli*	75 (43)	97.67%	90.70%		90.7%	I
Awosile et al. [73]	10.1139/cjm-2020-0442	2020	Canada	*E. coli*	144	65.28%	61.81%	21.53%	26.39%	S
Mollenkopf et al. [40]	10.1089/fpd.2017.2390	2018	USA	*E. coli*	497	11.10%	77.80%	17.51%	12.88%	S

Abbreviations: Pheno-ESBL/AmpC = phenotypic ESBL or AmpC resistance; Geno-ESBL/AmpC = genotypic ESBL or AmpC detection; Year = year of publication; Country = country where samples were collected; Denominator: S = sample-based prevalence (number of resistances per total number of samples); I = isolate-based prevalence (number of resistances per total number of isolates). ^a^ Combined prevalence for all listed *Enterobacteriaceae*.

**Table 6:** ESBL and AmpC resistance in meat samples (20–50%; seven studies).

Author	DOI	Year	Country	Bacteria	n Samples (Isolates)	% Pheno-ESBL	% Pheno-AmpC	% Geno-ESBL	% Geno-AmpC	Denominator
Abdallah et al. [74]	10.1371/journal.pone.0136052	2015	Egypt	KEC, *E. coli*	100 (106)	*Klebsiella* spp. 41.07%, *Enterobacter* spp. 11.61%, *Citrobacter* spp. 0.00%, *E. coli* 8.93%		65.09% ^a^		S
Sheikh et al. [75]	10.1089/fpd.2011.1078	2012	Canada	*E. coli*	206 (193)	28.15%		75.13%	25.24%	S
Moawad et al. [76]	10.1186/s13099-017-0206-9	2017	Egypt	*E. coli*	90 (15)	40.00%		66.67%		I
Kola et al. [77]	10.1093/jac/dks295	2012	Germany	*E. coli*, *Enterobacter* spp.	399	46.30% ^a^				I
Hadžić-Hasanović et al. [78]	10.17392/1206-20	2020	Bosnia and Herzegovina	*E. coli*	100 (64)	29.00%		24.00%		S
Randall et al. [79]	10.1111/jam.14687	2020	UK	*E. coli*	622	2016: 45.00%, 2018: 13.60%	11.25%	18.48%		S
Börjesson et al. [80]	10.3201/eid2204.151142	2016	Sweden	*E. coli*	368 (190)	8.42%	40.49%			S

Abbreviations: Pheno-ESBL/AmpC = phenotypic ESBL or AmpC resistance; Geno-ESBL/AmpC = genotypic ESBL or AmpC detection; Year = year of publication; Country = country where samples were collected; Denominator: S = sample-based prevalence (number of resistances per total number of samples); I = isolate-based prevalence (number of resistances per total number of isolates); KEC = *Klebsiella* spp., *Enterobacter* spp., and *Citrobacter* spp. ^a^ Combined prevalence for all listed *Enterobacteriaceae*.

**Table 7:** ESBL and AmpC resistance in meat samples (<20%; four studies).

Author	DOI	Year	Country	Bacteria	n Samples (Isolates)	% Pheno-ESBL (S/I)	% Pheno-AmpC (S/I)	% Geno-ESBL (S/I)	% Geno-AmpC (S/I)	Denominator
Ružauskas et al. [81]	n.a.	2010	Lithuania	*E. coli*	240 (100)	7%				S
Agersø et al. [82]	10.1093/jac/dkr507	2012	Denmark	*E. coli*	153	3.3%		35.75%	47.83%	S
Chenouf et al. [83]	10.1089/mdr.2020.0024	2020	Algeria	*E. coli*, *Klebsiella* spp.	136 (78)	*E. coli* 5.88%, *Klebsiella* spp. 3.58%		9.56% ^a^		S
Mollenkopf et al. [40]	10.1089/fpd.2017.2390	2018	USA	*E. coli*	497	11.1%	77.8%			S

Abbreviations: Pheno-ESBL/AmpC = phenotypic ESBL or AmpC resistance; Geno-ESBL/AmpC = genotypic ESBL or AmpC detection; Year = year of publication; Country = country where samples were collected; n.a. = no DOI available; Denominator: S = sample-based prevalence (number of resistances per total number of samples); I = isolate-based prevalence (number of resistances per total number of isolates). ^a^ Combined prevalence for all listed *Enterobacteriaceae*.

#### 2.2.5. Comparison of Different Farming Conditions

In nine studies, prevalence was determined as part of comparisons between broiler groups on different farming conditions (antibiotic-free, organic, conventional) or medical treatment management (treated versus non-treated). Conventional broiler systems generally showed higher resistance levels than organic or antibiotic-free farms, although some studies still found substantial resistance in all production types. In particular, two studies detected high percentages of resistant *Enterobacteriaceae* (50–100%): In The Netherlands, Stuart et al. [84] compared 38 organic and 60 conventional retail chicken breast samples and found that the prevalence of ESBL-producing microorganisms was 100% (95% CI: 94–100%) in the conventional samples, compared to 84% (95% CI: 70–93%) in the organic samples (*p* value < 0.001). The distribution of ESBL genes in the conventional and organic samples was 42% vs. 56% for CTX-M-1 (not significant), respectively, 20% vs. 42% for TEM-52 (not significant), and 23% vs. 3% for SHV-12 (*p* < 0.001). Additionally, CTX-M-2 (7%), SHV-2 (5%), and TEM-20 (3%) were detected exclusively in the conventional samples. In Canada, Chalmers et al. [85] investigated fecal samples from six antibiotic-free farms (60 samples) and seven conventional farms (58 samples), finding extended-spectrum cephalosporin (ESC)-resistant *E. coli* in 90% of the antibiotic-free samples and 95% of the conventional samples. Overall, both groups showed similar resistance levels. Among these ESC-resistant *E. coli*, CTX-M was identified in 25% of the isolates from conventional farms and 11% from antibiotic-free farms. CMY was more frequent in 76% of the conventional isolates and 89% of the antibiotic-free isolates. Two studies by Musa et al., both originating from Italy and comparing samples from antibiotic-free, organic, and conventional farms, showed results with prevalences of resistance between 20 and 50%. One investigated cloacal, environmental, cecal, and skin samples from farms and slaughterhouses [86], where cefotaxime resistance was detected in 36.2% of the samples from antibiotic-free farms, 29.3% from organic farms, and 51.7% from conventional farms. The odds of resistance were significantly lower on organic and antibiotic-free farms than on conventional farms and significantly higher in the samples collected at slaughterhouses than in those taken on the farms. The second study by Musa et al. [87] investigated cloacal swabs and skin samples from a slaughterhouse. Cefotaxime resistance was found in 23.7% of samples from antibiotic-free farms, 24.3% from organic farms, and 43.7% from conventional farms, with a statistically significant difference between farm types (*p* = 0.001). No significant difference was observed between the cloacal swabs and skin samples (*p* = 0.520).

Another two studies resulted in lower percentages of resistant isolates (<20%). Much et al. [88] compared 962 cecal isolates of organic and conventional farms in Austria (2010–2016) and revealed 2.0% ESBL producers in the organic samples and 1.9% in the conventional samples. Pesciaroli et al. [89] collected cecal samples from antibiotic-free, organic, and conventional farms, with prevalences of ESBL-producing *Enterobacteriaceae* of 4.8% in the antibiotic-free samples, 1.8% in the organic samples, and 3.4% in the conventional samples. Among these, 76% carried CTX-M-15, 12% TEM, and 8% SHV genes.

Three additional studies focused on different objectives. Voets et al. [90] compared plasmid-mediated Ampicillinase C beta-lactamase (pAmpC) genes between chicken meat and human clinical isolates in The Netherlands, finding that 12% of the meat isolates carried pAmpC, specifically CMY-2. Baron et al. [91] analyzed fecal samples from French broilers treated with third-generation cephalosporins and non-treated broilers, finding that the treated group had a significantly higher phenotypic prevalence of ESBL-producing bacteria than the non-treated group. Genotypic analysis revealed the presence of CTX-M in 14.3% of the isolates, TEM in 4.9%, and CMY-2 in 5.6%. In an experimental study in Canada, Merchant et al. [92] investigated the effect of feed supplementation on *E. coli* in litter across six different diets. The results showed significant treatment effects, with ceftiofur resistance ranging from 11.1% to 44.6%.

#### 2.2.6. Wastewater

Among the four European studies on poultry slaughterhouse wastewater, the extractable data were limited but indicated substantial ESBL resistance, ranging from a moderate range in Spanish wastewater to very high cefotaxime in German process and wastewater samples. In particular, Sabaté et al. [93] analyzed four wastewater samples in Spain and found that 21% were ESBL-producing. Facciola et al. [94] investigated wastewater from 18 Italian slaughterhouses, identifying beta-lactam resistance in *E. coli*, *Citrobacter* spp., and *Enterobacter* spp. without specifying the number of poultry-related samples.

A comprehensive study by Savin et al. [95] in Germany examined the process and wastewater from two slaughterhouses. Among 186 *E. coli* isolates, 99% in slaughterhouse 1 and 86% in slaughterhouse 2 were resistant to cefotaxime. Additionally, over 90% of the 71 isolates from the KEC group were cefotaxime-resistant. Two further publications from the study included smaller datasets and are only briefly mentioned [96,97]. Homeier-Bachmann et al. [98] studied wastewater from three poultry slaughterhouses in Germany, but no extractable results were available.

#### 2.2.7. Studies Investigating Only *Klebsiella* spp.

Across the five studies focused on resistant *Klebsiella* spp., ESBL-positive strains were very common, and several studies represented nearly all recovered isolates, even though only one study aimed to determine prevalence. Specifically, Ramadan and Awad [99] aimed to calculate prevalence and found 48.8% ESBL-producing *Klebsiella* spp., 84.6% carried *bla*_TEM_, and 30.8% *bla*_CTX-M_. Daehre et al. [100] did not aim to determine prevalence, but were able to isolate 41 *Klebsiella* spp. strains from various stages of the broiler production chain, all of which carried the ESBL gene *bla*_SHV-2_. One study by Savin et al. [101,102] examined process and wastewater, identifying 63 PCR-confirmed ESBL-producing *Klebsiella* spp., representing 88.8% of the isolates. Two studies from Egypt reported the presence of beta-lactamase-producing *Klebsiella* spp. Elmonir et al. [103] investigated diseased chickens and detected 19 PCR-confirmed resistant *Klebsiella* spp. out of 160 organ and environmental samples. Abdallah et al. [74] found that all *Klebsiella* spp. isolates from retail meat were ESBL producers.

#### 2.2.8. Other Targets

Four studies reported genotypic findings following PCR confirmation of only ESBL-positive isolates, showing moderate to high carriage of ESBL genes. In Canada, Ghosh et al. [104] identified ESBL-positive *E. coli* in 12.7% of broiler flocks, with all positive isolates carrying CTX-M-1. In Tunisia, 24% of cloacal samples from five farms contained ESC-resistant *Enterobacteriaceae*, with considerable variation between farms (ranging from 4% to 67%). Among these, 98% harbored CTX-M, 10% carried SHV-12, and 2% CMY-2 [105]. While another study by Hassen et al. [106] in Tunisia showed that 31.5% of isolates harbored ESBL genes. In Germany, Irrgang et al. [107] reported that 25.9% of 567 *E. coli* isolates from broiler production carried resistance genes, with 22.2% testing positive for SHV.

Across nine studies with other primary objectives, ESBL/AmpC-producing *E. coli* were repeatedly detected in various environmental compartments around broiler production (air, slurry, surfaces, flies, manure) and even in farm workers. In more detail, in Slovakia, Gregova et al. [108] examined bioaerosols and surface swabs in a poultry slaughterhouse and found that 43% were ESBL-producing *E. coli*. Among 19 selected isolates, 16 carried CMY-2, while no CTX-M was detected. In Spain, Solá-Ginés et al. [109] collected 682 flies from five poultry farms and detected ESBL-producing *E. coli* in 6.2%. Among the 42 positive isolates, 54.8% carried CTX-M-1, 43% CTX-M-14, and 2.4% CTX-M-9. Additionally, 78.6% carried TEM; SHV and CMY-2 were undetected. In Germany, Laube et al. [110] collected samples from the environment in seven broiler farms. Slurry samples were positive in 12 of 14 cases, and 28.8% of the surrounding boot swabs contained resistant *E. coli*. Three of the forty air samples outside the barns tested positive. Of 51 ESBL/AmpC-producing *E. coli*, 82% carried ESBL or CMY. Genetic analysis suggested transmission of resistant strains from the barn to the surrounding environment. In The Netherlands, Huijbers et al. [111] monitored cloacal and environmental samples from a broiler flock raised without antibiotics. A susceptible–infectious–susceptible (SIS) model demonstrated a rapid increase in ESBL-*E. coli* prevalence, reaching nearly 100% by day three. Martínez-Álvarez et al. [112] sampled manure and indoor air on a broiler farm in Spain, where 20.7% ESBL-producing *E. coli* were PCR-confirmed with SHV-12. Wadepohl et al. [113] studied stool samples from chicken farm workers and found that 5.1% carried ESBL-producing *Enterobacteriaceae*.

A total of 25 additional studies were reviewed and are presented in the Supplementary Material (Table S2). However, as these studies were assessed to be of lower methodological quality or lacked sufficient data, their findings were not considered in the main results section.

### 2.3. Carbapenem Resistance

Fifty-four studies investigated carbapenem resistance in the period from 2009 to 2022. Three studies came from North America, and eighteen were from North Africa. Samples from 33 studies were collected in Europe. Carbapenem resistance in broiler-related *Enterobacteriaceae* was overall very rare, with only a few findings in meat from Egypt, Algeria, and Spain.

Eleven studies analyzed samples from retail meat sources and rarely detected carbapenem-resistant bacteria. For instance, carbapenem-resistant *Klebsiella* spp. were found in chicken carcasses from Egyptian butcheries (26.1% phenotypically carbapenem-resistant *Klebsiella* spp. [74]) and in chicken meat from Algeria (16.0%). In Spain, a study reported that approximately 70% of *E. coli* isolates from chicken meat were carbapenem-resistant [114]. In contrast, several studies from North Africa and Europe did not detect carbapenem-resistant *E. coli* in meat samples (Randall et al. [70], Zarfel et al. [71], Chenouf et al. [83], Ramadan et al. [115], Gousia et al. [116], Pavlickova et al. [117]). Two additional investigations (Ojer-Usoz et al. [118], Sgariglia et al. [119]) addressed the same question but did not provide conclusive resistance data. Six of the seven studies that investigated samples collected from slaughterhouses did not show evidence of carbapenem resistance [46,51,55,61,65,78,89]. Concerning the studies that investigated fecal samples, only one study out of eight detected carbapenem-resistant *E. coli* (1.8%) in Egypt [32]. Regarding samples from diseased animals—mainly from North Africa—carbapenem-resistant *Klebsiella* spp. were found in two Egyptian studies (Elmonir et al. [103] with 40%; Hamza et al. [120] with 43%), while no such resistant pathogens were identified in the remaining three studies [43,45,121]. Comparison of conventional, organic, and antibiotic-free chicken production samples revealed only carbapenem-susceptible isolates [87,88]. Three studies investigated carbapenemases in wastewater; in two of these, no resistance was detected [95,98]. In the third, Facciola et al. [94] examined samples from various species; no carbapenem resistance was observed. Similarly, thirteen studies that focused exclusively on ESBL-positive isolates did not detect carbapenem resistance [24,27,31,48,63,64,67,69,73,105,122,123,124]. Two studies were excluded from the main results due to insufficient methodological quality [125,126].

### 2.4. Colistin Resistance

A total of 48 studies investigated colistin resistance. All of the studies were published within the timeframe of 2014 to 2022. Most of them (n = 32) were published between 2019 and 2022, while sixteen studies were published between 2014 and 2018. The majority of the studies were published in European countries (n = 30). The remaining studies originated from countries in North Africa (Egypt, Tunisia, Algeria).

#### 2.4.1. Prevalence Studies

Ten studies focused on determining prevalence, all of which reported prevalences of resistance below 20%. In more detail, five studies from different European countries were all conducted within a similar timeframe. These studies focused primarily on cecal or fecal samples and reported comparable percentages of resistant *Enterobacteriaceae*, consistently ranging between 1% and 5% [38,52,127,128,129]. In contrast, three separate studies, one from the UK and two from Algeria, reported negative results. Randall et al. [79] analyzed meat samples over a comparable period and did not detect colistin-resistant isolates. Similarly, Agabou et al. [126] and Messaili et al. [53], who investigated fecal and intestinal samples, also found no positive cases.

Moawad et al. conducted two studies in Egypt: In 2016, they analyzed 576 cloacal swab samples from poultry flocks, isolating 72 *Enterobacteriaceae* strains, of which 63 were *E. coli*. Five isolates (7.9%) were phenotypically resistant to colistin and carried the *mcr-1* gene [32]. In contrast, their 2017 study on 90 chicken meat samples yielded 15 *E. coli* isolates, all susceptible to colistin [76].

Two European studies by Casella et al. [64] and Päivärinta et al. [61] investigated meat samples, whereby both found no colistin resistance phenotypically and genotypically.

Three studies examined colistin resistance using cecal and intestinal swabs collected at slaughterhouses, none of which found any colistin resistance. Belmahdi et al. [55] tested 61 intestinal swabs in Algeria in 2014 and found that all isolates were susceptible to colistin. Similarly, Much et al. [88] reported no colistin resistance among 962 cecal isolates from Austria in 2010–2016. Likewise, Päivärinta et al. [61] investigated cecal and meat samples in Finland in 2015 but detected no colistin resistance genes.

#### 2.4.2. Comparison of Different Farming Conditions

Five studies compared resistance patterns between different treatment groups in healthy animals. Musa et al. [87] (compared conventional, organic, and antibiotic-free farms) and Much et al. [88] (investigated organic and conventional farms) found no resistant isolates, while Pesciaroli et al. [89] (samples from conventional, organic, and antibiotic-free farms) and Majewski et al. [130] (who investigated colistin-treated and non-treated fecal samples) reported a higher prevalence of resistance on conventional farms than in organic or antibiotic-free systems. Additionally, Ribeiro et al. [131] demonstrated that the number of PCR-positive *Enterobacteriaceae* decreased as the withdrawal period from colistin use increased.

#### 2.4.3. Clinical Samples

Six studies examined colistin resistance in diseased broiler chickens, yielding a lower prevalence of resistance (<20%) in four studies (two from Europe and North Africa). However, two studies found higher resistance levels (between 20 and 40%) in North Africa. More precisely, Mesa-Varona et al. [49] observed a colistin resistance of 5.7% among clinical *E. coli* isolates from cecal samples in Germany. In Poland, Majewski et al. [132] documented 15.3% colistin-resistant *E. coli* isolates and 15.5% in *Klebsiella* spp. isolates collected from various organs of affected birds. In Algeria, Halfaoui et al. [121] examined organ samples from 180 diseased chickens diagnosed with colibacillosis and identified 6.54% colistin-resistant *E. coli*. In Egypt, Elmonir et al. [103] examined 160 samples (100 organ and 60 environmental samples) and isolated 1.25% resistant *Klebsiella* spp. In contrast, Dhaouadi et al. [44] found 24% colistin-resistant isolates in broilers with colibacillosis from three farms, with more than half of the resistant isolates carrying the *mcr-1* gene. Even higher levels of resistant *E. coli* were reported by Badr et al. [133], who found that 41.1% of isolates from pooled organ samples across 120 farms were resistant, with all isolates carrying the *mcr-1* gene.

#### 2.4.4. Wastewater

Results from wastewater investigations were published in three studies conducted in Germany, where colistin-resistant *E. coli* and *Klebsiella* spp. were repeatedly found in wastewater from poultry slaughterhouses. In more detail, two separate studies by Savin et al. focused on poultry slaughterhouses. The first study [95] showed 9.8% phenotypic resistance to colistin, and PCR analysis confirmed resistance genes in 72%. Additionally, the study investigated isolates of a KEC group (*Klebsiella* spp., *Enterobacter* spp., *Citrobacter* spp.), detecting colistin resistance in 14.1%, with PCR performed on a subset of resistant *Klebsiella* spp. isolates, of which 50% were confirmed. The second study by Savin et al. [101], using samples from the same timeframe and locations, specifically analyzed *Klebsiella* spp. isolates and reported higher percentages of colistin resistance: 32.4% exhibited phenotypic colistin resistance. A third German study by Homeier-Bachmann et al. [98] investigated wastewater from three additional poultry slaughterhouses. Although the study reported colistin-resistant *E. coli* isolates, the lack of sufficient detail in the study design prevented the reliable extraction of specific prevalence data.

#### 2.4.5. Other Targets

Six studies examined only ESBL-positive isolates for colistin resistance, whereas five studies—three from Europe and two from North Africa—reported low prevalences of resistance (<20%). One Tunisian study detected higher percentages pheno- and genotypically. In detail, Vogt et al. [58] could not detect any colistin resistance in meat in Switzerland, followed by Kluytmans-van den Bergh et al. [134], who found 1.1% phenotypically resistant bacteria and 1.6% harboring *mcr-1* in meat samples in The Netherlands. Balázs et al. [28] detected 2.6% colistin-resistant ESBLs in feces from Hungary. The North African studies showed a 100% susceptibility in Algeria [126], compared to Saidani et al. [105], who reported 10% resistant *E. coli* and 0% in *Klebsiella* spp. In Tunisia, Hassen et al. [106] analyzed fecal and meat samples, detecting positivity of 53.1% and 66.7%, respectively, with 96.6% of the positive isolates carrying the *mcr-1* gene.

Four studies (three European and one North African) reported colistin resistance as an additional finding, although their primary focus was on other resistance patterns or targets, showing very low or no colistin resistance. Myrenås et al. [135] investigated colistin and cephalosporin resistance in cecal and meat samples and detected only 0.3% colistin-resistant *E. coli* isolates in Scandinavia. In Portugal, Manageiro et al. [124] analyzed ESBL-*E. coli* in cecal samples from poultry slaughterhouses and identified 3% reduced susceptibility to colistin. Schwaiger et al. [136] analyzed 438 isolates obtained from fecal samples of six broiler chicken flocks in Germany and reported a decrease in colistin resistance from 4.8% in week three to 0.9% in week five. Chaalal et al. [137], who studied meat samples in Algeria primarily for carbapenemase-producing bacteria, detected no colistin-resistant isolates at all.

Eight studies that reported colistin resistance were reviewed and are presented in the Supplementary Material (Table S4). However, their findings were not included in the main Results Section due to lower methodological quality or incomplete data reporting.

### 2.5. Fluoroquinolone Resistance

Ninety-two studies investigated fluoroquinolone resistance, which were published in the period from 2006 to 2022 (fifty-three studies from 2016 onward). In 57 studies, samples were collected from Europe, 24 from North Africa (Egypt, Algeria, and Tunisia), and 11 from North America.

#### 2.5.1. Prevalence Studies

Eleven studies aimed at determining the phenotypic prevalence of ciprofloxacin- and enrofloxacin-resistant *Enterobacteriaceae*, with seven from Europe, two from North Africa, and two from North America (Table 8). Overall, the Europe and Norboulth Africa studies showed moderate to high prevalences in fecal, cecal, liver, and meat samples, whereas an Iceland study presented lower levels, and the North American studies detected no resistance in comparable samples.

#### 2.5.2. Studies with Non-Prevalence Primary Objectives

Most studies (65 studies) focused on aims other than prevalence estimation, examining ciprofloxacin- and enrofloxacin-resistant *Enterobacteriaceae* across Europe, North America, and North Africa.

##### Europe

Across Europe, fluoroquinolone resistance in broiler-related *Enterobacteriaceae* in 21 studies was highly variable (Table 9), ranging from absent or low levels in some countries and different sample types to very high resistance levels in others (e.g., Poland and Romania), with several studies indicating higher resistance in conventional compared with organic or antibiotic-free (ABF) production systems.

##### North America

Fluoroquinolone resistance in seven North American studies was absent or extremely low. E.g., no resistance was found in two studies that investigated fecal samples [33,54], 1% was reported by Vounba et al. [138], and 0.28% was reported by Varga et al. [139]. Meat samples from Canada showed similar results, with no observed ciprofloxacin resistance [75]; three ciprofloxacin-resistant isolates reported by Awosile et al. [73], without mentioning the number of analyzed isolates; and just 0.2% in carcass samples [37].

##### North Africa

Six North African studies reported consistently high fluoroquinolone resistances in several sample types from Egypt and Algeria besides variable results from Tunisia. In particular, 43.3% of ciprofloxacin-resistant *E. coli* out of 120 isolates of meat samples could be observed in Egypt [115]. No resistance was found in one Tunisian study that investigated fecal samples [140], but another Tunisian study by Abbassi et al. [31] found 32.5% ciprofloxacin-resistant *E. coli* in fecal samples, analyzing only multi-resistant isolates. An Algerian study showed higher prevalences of resistance, with 89% in ciprofloxacin in fecal samples [141]. Hamed et al. [142] observed 71% in organ samples and up to 58.6% in cloacal, skin, and environmental samples. Similar to the findings of Hamed et al. [142], another Egyptian study revealed 72.9% ciprofloxacin-resistant *Klebsiella* spp. in organ and environmental samples [103].

##### Wastewater

Three studies investigated wastewater from poultry slaughterhouses. Two of these studies originated from Germany, reporting 53% ciprofloxacin-resistant *E. coli* and 15.5% ciprofloxacin-resistant *Klebsiella* spp./*Enterobacter* spp./*Citrobacter* spp. (KEC) in one study [98] and 1.1% ciprofloxacin-resistant *E. coli* in the second German study [98]. Sabaté et al. [93] found a ciprofloxacin resistance of 56% in *E. coli* isolates from Spain.

##### Clinical Samples

Eight studies investigated isolates of diseased broilers (mostly colibacillosis), whereby the studies from Europe and the USA reported very low to modest levels of fluoroquinolone resistance, whereas the North African studies consistently showed very high prevalences. In detail, one study from Germany detected 20% of 20 ESBL-positive isolates as enrofloxacin resistant [48]. Huang et al. [47] revealed 3.4% enrofloxacin-resistant *E. coli* from clinically ill animals out of laboratories in several US states. In North Africa, two Egyptian studies showed 82% ciprofloxacin-resistant *E. coli* [45] and 75% enrofloxacin-resistant *E. coli* [83]. Another Egyptian study investigated clinical and non-clinical isolates (100% enrofloxacin-resistant non-clinical *E. coli*) [143]. Dhaouadi et al. [44] analyzed 100 organ samples from colibacillosus-diseased animals and found 68% fluoroquinolone-resistant *E. coli*. The highest prevalence of enrofloxacin resistance was observed in Algeria, at 86.3% [121]. Badr et al. [43] found 66.1% phenotypic ciprofloxacin resistance in organ samples.

##### Other Targets

Across thirteen studies restricted to ESBL-*Enterobacteriaceae*, fluoroquinolone resistance varied widely in Europe, ranging from very low to about 50%. In contrast, the North African studies showed very high levels. In detail, Vogt et al. [69] found a low prevalence of 1.4% in ESBL-positive isolates from retail chicken meat in Switzerland. Kola et al. [77] and Belmar Campos et al. [67] reported 7.6% and 23% ciprofloxacin resistance among ESBL-producing *Enterobacteriaceae* from chicken meat in Germany. Smet et al. [22] found 8.7% enrofloxacin resistance in cloacal samples from broilers on five Belgian farms. Stuart et al. [84] reported 14% ESBL-producing *E. coli*. Blanc et al. [17] found 31.2% ciprofloxacin resistance among 192 ESBL-producing isolates from fecal samples in Spain, while Machado et al. [144] observed 50% resistance among 14 ESBL-producing *Klebsiella* spp. from meat and feces in Portugal; however, both of these studies were assessed as having poor methodological quality. Among the North African studies, Saidani et al. [105] and Hassen et al. [106] reported similarly high levels of resistance, with 86.0% of cloacal samples and 86.1% of ESBL-producing isolates from meat samples resistant to enrofloxacin or ciprofloxacin in Tunisia, respectively.

**Table 8:** Phenotypic fluoroquinolone resistance in eleven prevalence studies.

Author	DOI	Year	Country	Sample Type	Bacteria	n Samples (Isolates)	% Pheno-R	Denominator
Ramadan and Awad [99]	10.1186/s12941-016-0174-9	2016	Egypt	samples other than feces	*E. coli*	400 (116)	41.38%	I
Ceccarelli et al. [34]	10.1099/jmm.0.001176	2020	The Netherlands	feces	*E. coli*	(1811)	62.23%	I
Gousia et al. [116]	10.1089=fpd.2010.0577	2011	Greece	meat	*E. coli*	19 (8)	62.50%	I
Hanon et al. [38]	10.1016/j.prevetmed.2015.09.001	2015	Belgium	samples other than feces	*E. coli*	(1132)	>50.00%	I
Kaesbohrer et al. [145]	10.1111/j.1863-2378.2011.01451.x	2012	Germany	meat, feces	*E. coli*	(397)	meat: 53.10%, feces: 43.10%	I
Mainali et al. [146]	10.4315/0362-028X.JFP-13-203	2013	Canada	samples other than feces	*E. coli*	(600)	0.00%	I
Ružauskas et al. [81]	n.a.	2010	Lithuania	samples other than feces	*E. coli*	240 (100)	47.00%	I
Zhao et al. [147]	10.1128/AEM.07522-11	2012	USA	meat	*E. coli*	(2494)	0.00%	I
Manageiro et al. [124]	10.1016/j.ijfoodmicro.2017.10.007	2017	Portugal	samples other than feces	*E. coli*	680 (202)	90.60%	S, I
Chenouf et al. [83]	10.1089/mdr.2020.0024	2020	Algeria	meat	*E. coli*, *Klebsiella* spp.	136 (78)	60.20% ^a^	I
Thorsteinsdottir et al. [57]	10.1111/j.1863-2378.2009.01256.x	2010	Iceland	cecal, meat	*E. coli*	146 (185)	cecal: 18.20%, meat: 36.00%	I

Abbreviations: Pheno-R = phenotypic fluoroquinolone resistance; Year = year of publication; Country = country where samples were collected; n.a. = no DOI available; Denominator: S = sample-based prevalence (number of resistances per total number of samples); I = isolate-based prevalence (number of resistances per total number of isolates). ^a^ Combined prevalence for all listed *Enterobacteriaceae*.

**Table 9:** Phenotypic fluoroquinolone resistance in 21 European studies with non-prevalence primary objectives.

Author	DOI	Year	Country	Sample Type	Bacteria	n Samples (Isolates)	% Pheno-R	Denominator
Casella et al. [64]	10.1016/j.ijfoodmicro.2017.07.005	2017	France	meat	*E. coli*	48 (77)	20.80%	I
Egea et al. [122]	10.1016/j.ijfoodmicro.2012.08.002	2012	Spain	meat	*E. coli*	15	32.26%	I
Fetahagić et al. [28]	10.2478/aiht-2021-72-3560	2021	Bosnia and Herzegovina	feces	*E. coli*	108	10.67%	I
Geser et al. [27]	10.1186/1746-6148-8-21	2012	Switzerland	feces	*E. coli*	93	6.45%	I
Gregova et al. [108]		2012	Slovakia	processing plant of a slaughterhouse	*E. coli*	(48)	43.00%	I
Hricová et al. [148]	10.21101/cejph.a4328	2017	Czech Republic	bedding	*E. coli*	126 (126)	61.11%	S, I
Martínez-Álvarez et al. [112]	10.3390/antibiotics11040444	2022	Spain	air, manure	*E. coli*	111 (111)	38.70%	S, I
Musa et al. [87]	10.3390/ani10071215	2020	Italy	cloacal, skin	*E. coli*	(conventional 135, ABF 131, organic 140)	conventional 44.4%, ABF 20.6%, organic 23.6%	I
Zarfel et al. [71]	10.3390/ijerph111212582	2014	Austria	meat	*E. coli*	50	0.00%	S
De Koster et al. [19]	10.3390/antibiotics10080945	2021	Belgium, The Netherlands	feces	*E. coli*	779	84.81%	S
García-Béjar et al. [114]	10.3390/ani11113197	2021	Spain	meat	*E. coli*	30 (240)	ca. 25.00%	I
Randall et al. [70]	10.1093/jac/dkq396	2010	UK	cecal	*E. coli*	388	4.60%	I
Much et al. [88]	10.1016/j.prevetmed.2019.104755	2019	Austria	cecal	*E. coli*	1031 (962)	63.83%	I
Pesciaroli et al. [89]	10.1016/j.ijfoodmicro.2019.108391	2019	Italy	cecal, different production systems	*E. coli*	855 (854)	conventional 67.7%, ABF 42.8%, organic 45.2%	I
Maciuca et al. [51]	10.1089/mdr.2014.0248	2015	Romania	cecal	*E. coli*	127 (90)	87.79%	I
Myrenås et al. [135]	10.1016/j.vetmic.2017.11.015	2017	Norway, Sweden, Iceland	cecal, meat	*E. coli*	(319)	13.50%	I
Pavlickova et al. [117]	10.1080/03601234.2015.1011959	2014	Czech Republic	meat	*E. coli*	(75)	16.00%	I
Persoons et al. [30]	10.1089/mdr.2009.0062	2010	Belgium	cloacal, cecal, neck skin	*E. coli*, *Enterobacter* spp.	2249 (2076)	14.98% ^a^	I
Vanni et al. [149]	10.3382/ps.2013-03627	2014	Italy	feces, organ	*E. coli*	(235)	24.20%	I
Wasyl et al. [150]	10.3389/fmicb.2013.00221	2013	Poland	cloacal	*E. coli*	753 (682)	72.24%	S
Galler et al. [151]	10.3390/antibiotics10040466	2021	Austria	intestine	*E. coli*	100	0.00%	S

Abbreviations: Pheno-R = phenotypic fluoroquinolone resistance; Year = year of publication; Country = country where samples were collected; ABF = antibiotic-free production system; Denominator: S = sample-based prevalence (number of resistances per total number of samples); I = isolate-based prevalence (number of resistances per total number of isolates). ^a^ Combined prevalence for all listed *Enterobacteriaceae*.

#### 2.5.3. Comparison of Phenotypic and Genotypic Fluoroquinolone-Resistant *Enterobacteriaceae*

The findings revealed significant differences between the samples and phenotypic and genotypic prevalence rates.

In Europe, phenotypic and genotypic resistances were generally lower than in North Africa, though certain sample types exhibited higher resistances, particularly in bedding, skin, fecal, and manure samples (Table 10). Only one study from Italy reported similarly high phenotypic and genotypic resistance.

In North Africa, the studies consistently indicated higher levels of fluoroquinolone resistance (Table 11), often in the moderate to very high range. Genotypic confirmation was often lower than phenotypic resistance.

#### 2.5.4. PCR-Confirmed Fluoroquinolone-Resistant *Enterobacteriaceae*

Two studies conducted only genotypic detection (*qnr*, *qep*, *aac*(6′)-*Ib*) or reported only genotypic results. In the study by Savin et al. [102], water samples from two poultry slaughterhouses in Germany were tested for resistance in *Klebsiella* spp., including phenotypic detection. Although absolute numbers for phenotypic resistance were not provided in the publication, 3 out of 71 *Klebsiella* isolates were identified as carriers of PMQR genes (4%). Niero et al. [50] examined lesion swabs from broilers with colisepticemia and found PMQR genes in 4 of 98 *E. coli* isolates (4%).

### 2.6. Regional and Overall Occurrence of Genotypic Resistance Patterns

Across all regions, 67 studies (40 studies from Europe, 21 from North Africa, and only six from North America) provided data on resistance genes at the isolate level. Out of these, 49 articles reported ESBL resistance proteins (CTX-M, SHV, and TEM), with an overall occurrence of 24.7% (n = 2277/9210) positive isolates, while at the same time, 26 studies investigated the AmpC resistance protein CMY-2, including 3701 investigated isolates, which resulted in an overall prevalence of 26.6% (n = 616/3701). Six studies investigated carbapenem resistance (OXA, VIM, KPC, and NDM), with an overall resistance of 4.4% (n = 32/730). Seventeen articles performed molecular detection of colistin resistance to *mcr-1* in environmental and other sample types and found 4% (n = 258/8674) of isolates to be positive. Ten studies tested 974 isolates for PMQR proteins (*qnr*, *qep*, *aac*(6′)-*Ib*) and yielded an overall prevalence of 17.3% (n = 168/974).

**Figure 3:**
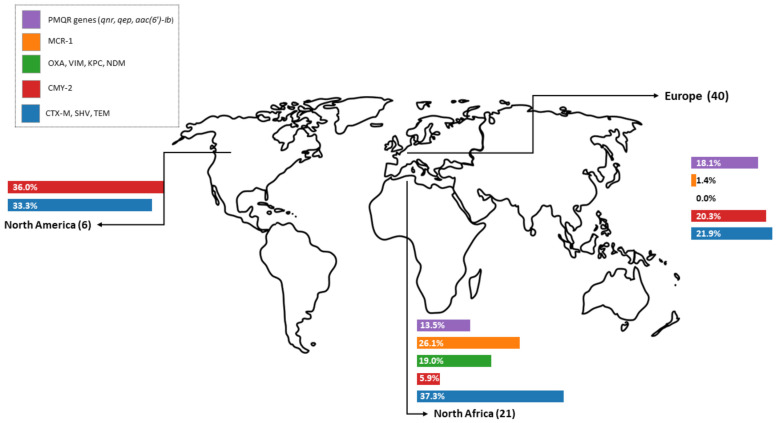
Prevalence of ESBL, AmpC, colistin, carbapenem, and fluoroquinolone resistance proteins in *Enterobacteriaceae* from 67 studies over the considered regions. Numbers of studies per region in brackets.

As shown in Figure 3, the highest occurrence of beta-lactam resistance genes was observed in North Africa, where 37.3% of isolates carried CTX-M, SHV, or TEM across 16 studies involving different sample types. In contrast, North America reported a higher prevalence of AmpC resistances (CMY-2), with 36% across six studies. Notably, carbapenem resistance genes were primarily reported from North Africa, where five studies identified them in 19% of *Enterobacteriaceae*, while one European study detected no carbapenemase genes. A similar pattern was seen for colistin resistance genes, with 26.1% in North Africa (six studies), no reports from North America, and only 1.4% in Europe (11 studies). Regarding PMQR proteins (*qnr*, *qep*, *aac*(6′)-*Ib*), six European studies found a prevalence of 18.1%, compared to 13.5% reported in four studies from North Africa.

### 2.7. Prevalence in Meat and Feces

#### 2.7.1. Negative Binomial Regression Model

**Figure 4:**
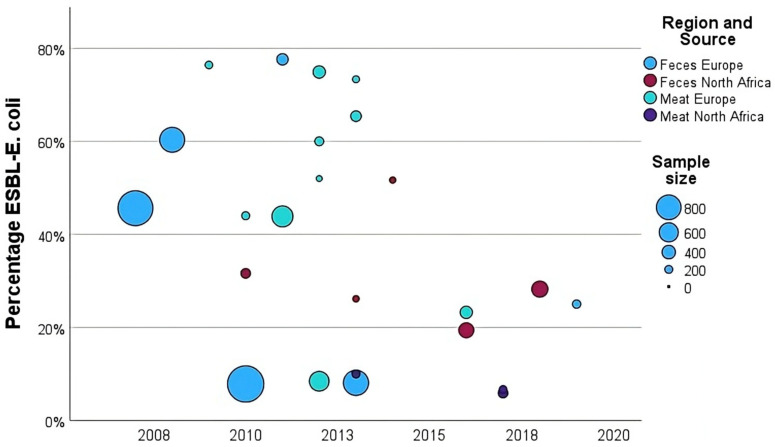
Prevalence of phenotypic ESBL-*E. coli* per region and year of publication (60 studies).

To explore how resistance patterns varied over time, between regions, and by sample types, Figure 4 shows the ESBL-*E. coli* prevalence reported in the studies included in the analyses of prevalence data. Studies reporting high prevalences mainly involved meat samples from Europe, before 2015, whereas studies from North Africa showed lower prevalences.

**Figure 5:**
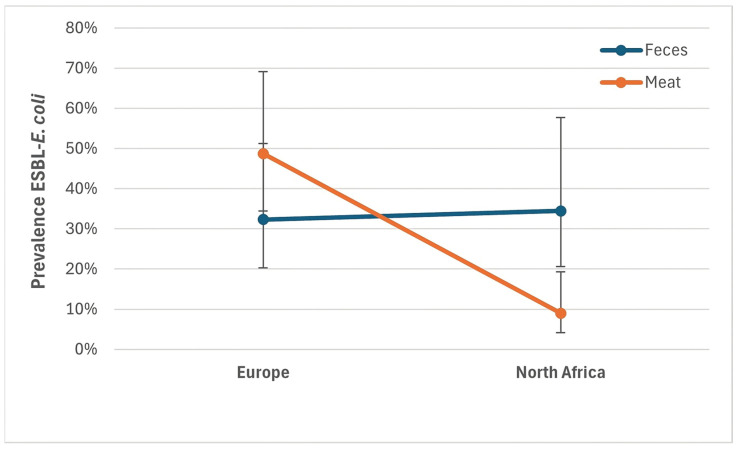
Maximum likelihood estimates and 95% CIs from a negative binomial regression model for region and sample type; 24 studies (16 from Europe, 8 from North Africa; 11 on feces, 13 on meat).

The regression model revealed that region and sample type had a significant influence on prevalence (global *p*-values: region *p* = 0.0118; sample type *p* = 0.0914; interaction *p* = 0.0030), while prevalence declined over time (IRR 0.93), but this effect was not statistically significant (*p* = 0.1228, Table 12). While there was no difference between Europe and North Africa in feces samples, meat samples in Europe had a significantly higher prevalence than in North Africa (*p* = 0.0029, Figure 5).

#### 2.7.2. Meta-Analyses

**Figure 6:**
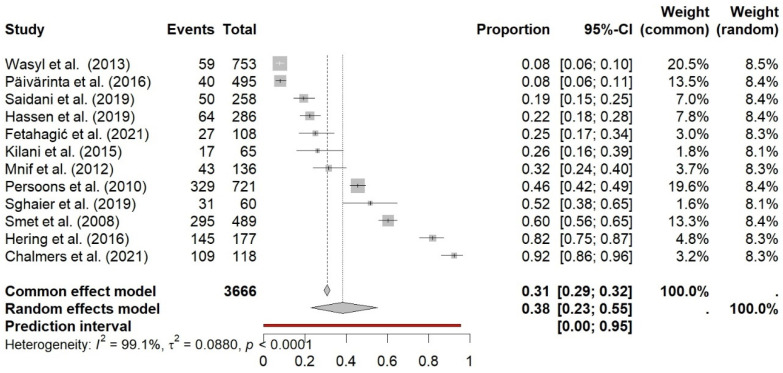
Forest plot showing the pooled prevalence (diamond) of ESBL-*E. coli* in broiler fecal samples from twelve studies [22,23,24,25,28,30,85,105,106,123,150,158].

**Figure 7:**
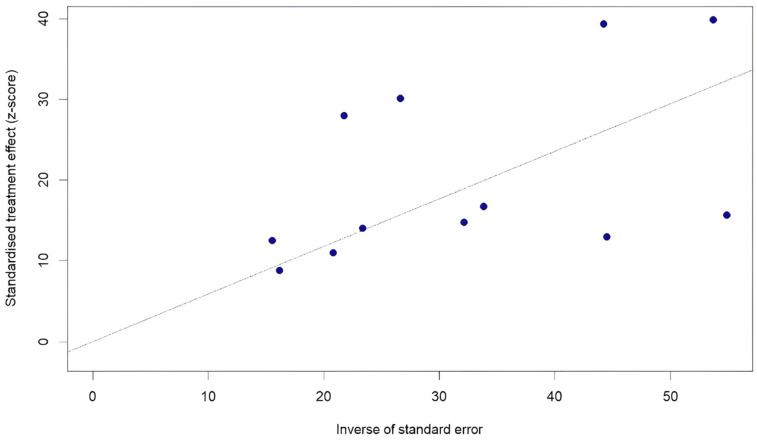
Galbraith plot showing the heterogeneity in twelve studies reporting the prevalence of ESBL-*E. coli* in broiler fecal samples.

The pooled prevalence of twelve publications about ESBL-resistant *E. coli* in fecal samples was estimated at 38% (95% CI 23–55%; I^2^ = 99.1%) (Figure 6). Neither the sample size nor the publication year seemed to impact prevalence. Six studies originated from Europe, five from North Africa, and one from North America. Between-study variability was high (between-study variance τ2 = 0.088, *p* < 0.0001), which corresponds to the Galbraith plot (Figure 7).

**Figure 8:**
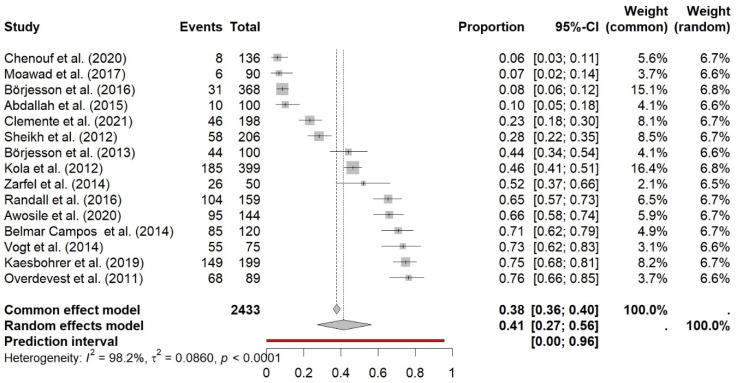
Forest plot showing the pooled prevalence (diamond) of ESBL-*E. coli* in broiler meat from 15 studies [65,66,67,68,69,70,71,73,74,75,76,77,80,83,159].

**Figure 9:**
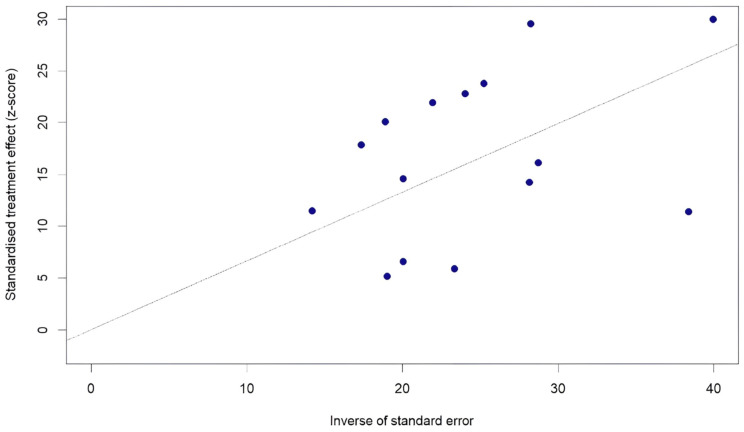
Galbraith plot showing the heterogeneity in fifteen studies reporting the prevalence of ESBL-*E. coli* in broiler meat samples.

Concerning the meat samples, the pooled prevalence was 41% (95% CI 27–56%; I^2^ = 98.2%) (Figure 8). The Galbraith plot also displays a large variability between the studies (Figure 9), which was statistically significant (τ2 = 0.086, *p* < 0.0001). Most studies originated from Europe (ten), followed by three from North Africa and two from North America.

## 3. Discussion

### 3.1. General

Our review included 6.7% of the originally selected articles. The data from 170 studies enabled us to calculate and compare the occurrence of phenotypic resistance and resistance genes in beta-lactams (ESBL and AmpC), colistin, carbapenemases, and fluoroquinolones in certain *Enterobacteriaceae* over a period of 21 years and three global regions. To our knowledge, this is the first study to use a systematic review and meta-analyses of prevalence data on ESBL-*E. coli* in fecal and meat samples, as well as the occurrence of resistance genes. In recent years, several reviews on specific resistances or on specific sample types were published (e.g., [160,161,162,163,164]), but no review covered the whole range of bacteria and resistance patterns covered in our publication.

As expected, this review comprised numerous studies including different sample types, different laboratory methods, articles from various countries, and a wide period of published articles. Corresponding to our results, Luz et al. [2] reported an increase in the number of publications on antimicrobial resistance of 450% between 1997 and 2018, reflecting the increased awareness of this topic. The most frequently used sample type in this review was fecal samples because they are easy to obtain and contain *Enterobacteriaceae*, which were our focus and represent the One Health aspect.

The detection method was a crucial factor contributing to the heterogeneity between studies. As mentioned by Silley et al. [165] in 2011, it is essential to distinguish between clinical and epidemiological cut-offs and to take both into account even in the context of monitoring. Clinical breakpoints guide antimicrobial therapy, but an isolate with reduced susceptibility may have sufficiently low minimum inhibitory concentration (MIC) to ensure clinical therapeutic success. Therefore, isolates with low susceptibility to an antimicrobial substance should be distinguished from those with clinical resistance.

A notable observation is the number of studies on the topic, yet very few focus on prevalence. Many studies appear to prioritize methods designed to maximize the detection of isolates rather than accurately assessing prevalence. Consequently, the methodologies employed in these studies are not well-suited for providing reliable prevalence estimates. The limited data availability in many countries can often be attributed to resource constraints, the absence of active surveillance programs, or a lack of transparency surrounding antibiotic use in livestock industries, further hindering a comprehensive understanding of resistance patterns. The lack of focus on epidemiological aspects is evident in many studies, as insufficient information was provided on sample sizes or the number of positive samples. In most cases, this limitation significantly hindered our ability to reliably calculate percentages of resistant bacteria, reflected in poor quality assessment scores and limited comparability across studies.

### 3.2. Beta-Lactam Resistance (ESBL- and AmpC-Producing Enterobacteriaceae)

Most studies focused on beta-lactam resistance, with a rise in publications after 2014, reflecting the increased attention to AMR. In our review, both fecal samples collected on farms and meat samples often showed high resistance patterns, with several studies reporting values between 50% and 100%. According to the latest European Union (EU) summary report, the presumptive prevalence of ESBL-/AmpC-producing *E. coli* in broilers and broiler meat was 34.9% and 29.2% at the Member state-group level in 2022 [166]. These surveillance data suggest lower average prevalences of resistance than many of the included studies, highlighting the importance of harmonized monitoring to provide representative estimates. However, when looking at individual countries, the EU data revealed heterogeneity, ranging from 3.4% in Denmark to 61.3% in Hungary [166]. This variation mirrors the broad range of resistance levels observed across the included studies in our review. This suggests that national differences in antimicrobial usage, biosecurity measures, and slaughterhouse hygiene may contribute to this variation.

In contrast, the results from slaughterhouse samples were more heterogeneous: half of the included studies reported resistance levels below 20%, while the other half ranged up to 100%, suggesting that factors such as sampling context, cross-contamination, and methodological differences may play a role. Samples obtained at slaughterhouses or from broiler meat are subject to additional influences, including cross-contamination during scalding and defeathering [167]. Foyle et al. [162] reported a median of 75% Gram-negative isolates resistant to cefuroxime in wastewater in 27 studies, which is higher than the results in our review. Regarding non-*E. coli* ESBLs, ESBL-producing *Enterobacter* spp. were most often detected in German wastewater, consistent with their ability to colonize the gastrointestinal tract. As other authors have described, clonally related strains were detected in the parent flock and later in the fattening flock, indicating pseudo-vertical transmission and/or cross-contamination [95,100].

Studies on diseased animals reported lower resistance rates than those in healthy broilers. One explanation could be that these studies were more targeted, with smaller sample sizes or restricted to specific clinical conditions, which may not represent the true extent of resistance in the general population.

Our review also highlights the importance of phenotypic testing and molecular methods. While many studies reported resistance based on phenotypic detection methods, fewer provided detailed genotypic data. The EU summary report emphasized that molecular characterization is required to determine whether transferable genes encoding extended-spectrum cephalosporin resistance are present [166]. Such information is essential to assess the potential for horizontal gene transfer and the dissemination of resistance patterns.

Overall, the findings highlight the persistence of ESBL- and AmpC-producing *Enterobacteriaceae* throughout broiler production and support the importance of coordinated monitoring strategies. Given the potential for transmission to humans through meat consumption and environmental exposure, reducing beta-lactam resistance in poultry remains a crucial target for global One Health approaches.

### 3.3. Carbapenem Resistance

The limited number of studies on carbapenem resistance highlights a significant gap in research on this critical antibiotic class. Fifty-four studies were identified, with a notable geographic bias: thirty-three were conducted in Europe, eighteen in North Africa, and just three in North America. This uneven distribution suggests an underrepresentation of data from many regions, notably where carbapenem use may differ significantly. Carbapenem resistance was rarely detected in chicken-associated *Enterobacteriaceae*, yet isolated studies reported alarmingly high percentages of carbapenem-resistance, particularly in *Klebsiella* spp. from North Africa and in one European study from Spain. While these findings may indicate true resistance hotspots, they could also reflect methodological differences or limited sample sizes. This highlights the need for harmonized surveillance systems to distinguish sporadic detections from emerging regional trends reliably. These findings may reflect differences in antibiotic usage practices. In North Africa, unregulated or excessive use of carbapenems in both human and veterinary medicine could drive the emergence and spread of resistance. The higher prevalence of resistance observed in North Africa may also be linked to inadequate surveillance systems, weak regulatory enforcement, and a lack of standardized guidelines for antimicrobial use [168]. This contrasts with Europe and the USA, where stricter antibiotic stewardship programs and bans on certain antibiotics exist. Our findings on carbapenem-resistant *Enterobacteriaceae* (CRE) align with those of Köck et al. [163], who also reported CRE in Algeria. In contrast, their review included two extensive European studies indicating that CRE were not highly prevalent in livestock. This supports the notion of significant regional differences in CRE prevalence, likely influenced by antibiotic usage patterns and regulatory practices. In addition, carbapenem resistance has also been reported in other bacterial species and production settings, such as carbapenem-resistant *Acinetobacter baumanii* in raw milk from dairy farm animals, highlighting that multiple livestock sectors may contribute to its emergence and spread [169]. The limited data, regional disparities in prevalence, and varying detection methods underscore the need for more comprehensive and standardized research on carbapenem resistance.

### 3.4. Colistin Resistance

Research on colistin resistance in broiler production has intensified in recent years; the first study included in this review was conducted in 2014. This timing matches the global discovery of the transferable *mcr-1* gene, which led to more monitoring [170]. Interestingly, we could not include studies on colistin resistance from the USA and Canada. One reason might be that the oral administration of colistin in livestock was never authorized in these countries [160]. In contrast, in North Africa, colistin is used widely even without a prescription [137], which is reflected by the high prevalence of colistin resistance at the phenotypic and molecular levels in our review. In Europe, the overall occurrence of colistin resistance was relatively low, reflecting the restricted or minimal veterinary use of colistin [161]. Some exceptions exist, such as the study from Poland that reported resistance rates around 15.8% [130], which may be linked to more frequent usage in that country. The studies by Badr et al. [133] and Dhaouadi et al. [44] suggest that diseased animals have higher resistance rates than healthy ones. The studies that compared production systems indicated that conventional farms sometimes showed higher resistance than organic or antibiotic-free systems [86,88,89,139]. While the number of such studies is limited, these observations indicate that farming practices and antibiotic usage can shape the occurrence and dissemination of colistin resistance [170].

### 3.5. Fluoroquinolone Resistance

Fluoroquinolones were classified as “highest priority critically important” antibiotics in human medicine besides third- and fourth-generation cephalosporins, macrolides, glycopeptides, and polymyxins [171]. Research on fluoroquinolone resistance in broilers shows an increase in the number of publications since 2016. Roth et al. [172] reported a rise in ciprofloxacin resistance in Spain from 17% in 2001 to 91% in 2016, potentially linked to the continued use of enrofloxacin in poultry, as its use is not entirely prohibited under EU directives [145].

In Europe, fluoroquinolones remain approved for treating bacterial infections in poultry, despite the 2006 ban on antibiotic growth promoters. In contrast, the Food and Drug Administration banned enrofloxacin use in the US as early as 2005 [172]. These policy differences may explain the significant disparities in resistance patterns between regions. North American studies consistently reported low or no resistance, whereas the prevalence in Europe reached up to 90%. This is consistent with EU monitoring data, which reported very high median ciprofloxacin and nalidixic acid resistance in broilers and high resistances in turkeys [167]. In North Africa, resistance data varied widely, which may reflect inconsistent antimicrobial use and surveillance systems in the region. Factors such as insufficient regulatory enforcement, lack of standardized treatment guidelines, and inadequate border controls exacerbate the problem [173]. Our findings indicate substantial variability in resistant isolates, with high numbers observed in the studies from Egypt and no resistance reported in the studies from Tunisia.

The prevalence of resistance observed in the studies should be viewed critically due to differences in detection methods and the number of isolates tested. Prevalences also showed wide variation across sample types. Notably, Italian meat samples [63] and Egyptian organ samples [42] showed close alignment between phenotypic and genotypic resistance, suggesting that PMQR genes might significantly contribute to phenotypic resistance in certain sample types. These differences may be shaped by alternative mechanisms (e.g., efflux pumps, target mutations) or environmental factors. In North Africa, high phenotypic resistance appears frequently even without associated PMQR genes, implying that non-genotypic factors could play a substantial role.

### 3.6. Regional and Overall Occurrence (Genotypic)

The fact that so many studies have been conducted in North African countries, in particular, may be because the prevalence of resistance is so high there. One of the reasons for this might be that even critically important substances are still used there as growth promoters [173]. Our genotypic findings underline this pattern, with the highest prevalence of ESBLs, carbapenemases, and colistin resistance genes reported from North Africa, in contrast to lower levels in Europe and North America, represented by only a few studies. Overall, ESBL and AmpC genes were the most frequently detected resistance determinants (approximately 25–27% of isolates), carbapenemase and colistin genes remained rare (around 4% each), and PMQR genes accounted for 17%. Notably, North Africa showed consistently higher prevalence across multiple gene classes, including ESBLs, carbapenemases, and colistin genes. At the same time, AmpC (CMY-2) was most frequent in North America and Europe, showing moderate ESBL/AmpC but more PMQR genes. However, these results should not be interpreted as representative prevalence estimates for each region, but rather as a synthesis of the available study data, which may be influenced by sampling strategies, isolate selection, and publication bias.

### 3.7. Analyses of Prevalence Data

As shown in Figure 4, most European studies before 2015 reported high levels of ESBL-producing *E. coli* in broiler fecal and meat samples, frequently exceeding 40–60%. After 2015, fewer studies were available, and the reported resistance appeared more heterogeneous, with some dropping below 20%. This shift may reflect the impact of EU measures restricting the off-label use of extended-spectrum cephalosporins in poultry production in 2012 [174]. Our regression model revealed that region and sample type significantly influenced the prevalence of ESBL-producing *E. coli*, while no consistent temporal decline could be confirmed. This shows that regional and methodological differences may substantially affect more than time trends. Notably, a higher prevalence was observed in European meat samples than in North African meat samples, while no regional differences were observed in fecal samples. Here, the possibility of publication bias must be considered (studies with higher resistance levels are more likely to be published), which plays a role in the variability [175].

#### Meta-Analyses

Through the meta-analysis of meat samples, the highest estimated ESBL-*E. coli* prevalence was 41%. The meta-analysis revealed that the average prevalence in meat samples was slightly higher than in fecal samples (38%). As reported by Projahn et al. [167], the effect of specific intervention steps in a slaughterhouse cannot prevent the spread of *Enterobacteriaceae* between carcasses and thus leads to an increase in prevalence. It has also been described several times that the structure of chicken skin has an influence on the adhesion of bacteria and that firmly attached bacteria are more difficult to remove during the plucking process [176]. However, the extremely high I^2^ values in both meta-analyses indicate considerable heterogeneity between studies and limit the interpretability of these pooled estimates. Our analysis of prevalence data, combined with Figure 4 and the generalized linear regression model, as described above, provides more nuanced insights into how prevalence varies over time, by region, and by sample type than the pooled estimates alone.

Only studies analyzing one colony per sample from healthy animals without preselection for multidrug resistance were included for fecal samples to avoid overestimating prevalence. Studies with more than one colony per sample were also considered for meat samples, because otherwise, too few studies would have been available. Nevertheless, analyzing only one colony per sample is most suitable for epidemiological prevalence studies, while testing multiple colonies can be helpful in laboratory diagnostics to show a broader picture of resistance. The lack of studies applying such epidemiological criteria suggests a possible publication bias, focusing on reporting higher resistance levels instead of representative prevalence estimates. In this context, classical statistical tests for publication bias, such as funnel plots and Egger’s test, are designed for comparative effect measures. In our single-group, prevalence-based meta-analyses with only a few studies and considerable heterogeneity, the results would be difficult to interpret and potentially misleading.

It was also surprising that even current studies reported high resistance levels, although the EU summary report on antimicrobial resistance shows a continuous decline i ESBL-*E. coli* prevalence in all European countries for many years [166], which may at least partly be explained by publication bias. Apart from that, the variance between the study results in both meta-analyses was surprising, considering the strict inclusion criteria of the studies. Publications in peer-reviewed journals focus on objectives other than representative prevalence estimates. This is a pity because national monitoring systems are not always available in English, and thus (except for the EU summary report), comparability of results from different nations is limited. We recommend publishing the results of (national) monitoring systems in peer-reviewed journals, thus making them available to the international research community.

For instance, the 2013 study by Koga et al. showed a prevalence of 65.4% in chicken carcasses [177] from Brazil, the world’s largest exporter of chicken meat and second-largest producer. This is similar to our results, but there is a lack of further studies in this area from Brazil. The fact that neither country, year, nor sample size affected the prevalence in feces and meat samples was surprising. Despite thorough inclusion criteria concerning high-quality scores and the study objective, homogeneity in the study results could not be achieved. The interpretation of study results should therefore be performed carefully. One possible explanation is that many studies covered the inclusion criteria but did not focus on prevalence. This leads us to conclude that the methods are designed to find many isolates, but this does not result in a reliable prevalence statement.

### 3.8. Conclusions

In conclusion, this systematic review and meta-analysis revealed substantial variation in prevalence and underscores the need for standardized surveillance systems and robust study designs. In addition, potential drivers, such as climate change and its impact on AMR in broiler production systems, should be considered. Future studies should adopt a clear epidemiological focus and comprehensive reporting of all baseline data, including sample sizes and the number of positive samples. This would allow for accurate prevalence calculations and improve the overall quality and comparability of research in this field.

## 4. Materials and Methods

This systematic review followed the guidelines of the Preferred Reporting Items for Systematic Reviews and Meta-Analyses (PRISMA) [178] and was registered in PROSPERO (International Prospective Register of Systematic Reviews) under the registration number CRD42024423479. The review question was “What is the extent of antimicrobial resistance in poultry farms and their environment?”

### 4.1. Inclusion and Exclusion

We included observational studies that reported the occurrence of antimicrobial resistance in broiler chicken production. We focused on resistance caused by beta-lactamases (ESBLs and AmpC) and carbapenemases, as well as colistin resistance and fluoroquinolone resistance in *E. coli*, *Klebsiella* spp., *Enterobacter* spp., and *Citrobacter* spp. The initial search string included *Shigella* spp., but no articles that examined *Shigella* spp. met the inclusion criteria. Therefore, *Shigella* spp. was not considered further in this review. This study was limited to publications between January 2002 and July 2022 and included studies from Europe, defined geographically, North America (USA and Canada), and North Africa. Accordingly, we excluded studies with samples from hatcheries and hatching eggs and samples from parent birds, breeders, and laying hens. Only peer-reviewed original research articles were included, while short communications, reviews, and other articles were excluded.

### 4.2. Search Strategy

PubMed (National Library of Medicine, Bethesda, MD, USA) and Web of Science (Clarivate Analytics, Philadelphia, PA, USA) were searched for studies published within the mentioned time frame. The literature search in the databases was carried out on the 4th of August 2022. The databases were searched using the following search strategy: “((chicken or poultry or broiler) AND (“*E. coli*” or *Escherichia* or *Enterobacter* or *Klebsiella* or *Shigella* or *Citrobacter*) AND (ESBL or AmpC or pAmpC or “plasmid-mediated AmpC β-lactamase” or “plasmid mediated AmpC β-lactamase” or “plasmid-mediated AmpC beta lactamase” or “plasmid mediated AmpC beta lactamase” or “plasmid mediated AmpC beta lactamase” or “extended-spectrum β lactamase” or “extended-spectrum β-lactamase” or “extended-spectrum beta lactamase” or “extended spectrum beta lactamase” or “colistin -resistance” or “colistin resistance” or “mcr-1” or mcr or “fluoroquinolone resistance” or “Fluoroquinolone-resistance” or “quinolone resistance” or “quinolone-resistance” or “carbapenem resistance” or carbapenemase or “carbapenemase producing” or PMQR or “Plasmid-mediated quinolone resistance” or “Antibiotic resistance” or “Antimicrobial resistance” or AMR or susceptibility or “Multi-drug resistance” or ABR or “Inhibitory effect” or “Multidrug resistance” or ARB or “Antimicrobial resistance bacteria” or MDR))”.

### 4.3. Study Selection

The software Rayyan (Rayyan Systems Inc., Doha, Qatar) was used for the screening process. For the study selection process, three reviewers (JK, DV, AA) screened the publications found through the title and abstract search strategy, following the inclusion criteria in Table 13. Possible duplicates were removed after manual confirmation. After the first screening, the reviewers’ decisions were unblinded. Reviewer disagreements were resolved by reaching a consensus.

### 4.4. Data Extraction from Eligible Studies

The publications that met the inclusion criteria by screening the titles and abstracts were collected in Citavi (Swiss Academic Software GmbH, Wädenswil, Switzerland). The data were extracted from the eligible studies selected by full-text reading and recorded in Microsoft Excel^®^ (Microsoft Corporation, Redmond, WA, USA). Full-text reading to assess eligibility and data extraction was conducted jointly by two reviewers (JK, RM). Studies were considered as eligible if the aim of the study was fulfilled by testing relevant chicken sample types (fecal, meat, environmental, or other) for the resistance types of interest (ESBL, AmpC, carbapenemases, colistin, fluoroquinolone) and reporting phenotypic and/or genotypic outcomes. Only observational studies were eligible.

Automation tools were not used, and contacting the study authors was not necessary. We collected general data concerning the continent and country from which the samples were collected. The samples were categorized as described above, and the bacteria and resistance patterns were noted. We recorded the laboratory methods and documented whether diseased or healthy animals were sampled.

The outcomes were the number of samples per sample type, the number of isolates, the number of phenotypically resistant isolates, the number of PCR-confirmed isolates, and the number of different detected genes. We considered the following genes: *bla*_CTX-M_, *bla*_SHV_, and *bla*_TEM_ for ESBL, *bla*_CMY-2_ for AmpC, *bla*_OXA_, *bla*_KPC_, *bla*_NDM_, and *bla*_VIM_ for carbapenemases, *mcr-1* for colistin resistance, and *qnr*, *qep*, and *aac*(6′)-*Ib* for PMQR.

### 4.5. Data Analysis

For all resistance types, the resistance patterns reported in the original publications were extracted when available; if not explicitly stated, the percentage of resistant bacteria was calculated based on the number of tested samples and, if given, the number of isolates, according to the study design. When genotypic results were reported, they were included alongside phenotypic findings. Calculations could not be performed if the data were unavailable or multiple sample types were included in the results and not presented separately. Such studies were excluded from the data analysis but were still mentioned. Supplementary Tables S1–S6 summarize the relevant studies for each resistance type included in this review and the studies that are mentioned but whose results were not reported due to poor methodological quality. These tables provide details on study characteristics (year, country, sample type, bacterial species, number of samples, and isolates) and phenotypic and genotypic resistance patterns.

The studies with similar objectives were grouped (e.g., sample type, aim of prevalence determination, study design, diseased animals). The results were further stratified within each region (Europe, North America, North Africa) into resistance level categories of high (50–100%), moderate (20–50%), or low (<20%). For fluoroquinolone resistance, the studies were analyzed in three groups: those reporting only phenotypic results, those presenting phenotypic and genotypic results in comparison, and those reporting exclusively genotypic results.

### 4.6. Regional and Overall Occurrence of Genotypic Resistance Patterns

Regional and overall occurrence of genotypic resistance patterns were calculated using only those studies that reported both the total number of isolates tested for specific resistance genes and the number of positive detections. Supplementary Table S5 summarizes the included studies. The studies were assigned to three geographic regions (Europe, North Africa, North America) for each resistance type: beta-lactam (ESBL and AmpC), carbapenemases, colistin resistance, and plasmid-mediated quinolone resistance (PMQR). A base world map was obtained from the Pixabay image repository (https://pixabay.com, image ID 160811, accessed on 11 August 2025) and edited in Microsoft PowerPoint^®^ (Microsoft Corporation, Redmond, WA, USA) to highlight the study regions and to insert genotypic resistance charts.

### 4.7. Analyses of Prevalence Data

To compare study results between regions and over time, we selected studies that investigated ESBL-resistant *E. coli* isolates in two sample types. Studies reporting on fecal or cloacal samples were included if they (1) investigated only one colony per sample, (2) examined only healthy animals, (3) had a minimum sample size of 50, and (4) did not restrict their analysis to multi-resistant isolates. The inclusion criteria for the analysis of meat samples were analogous; however, due to the limited number of eligible studies, those that examined more than one colony per sample were also included. This resulted in 12 (fecal) and 15 (meat) studies (Figure 6 and Figure 8). A figure to display the raw prevalences per study, taking the sample size into account, was constructed using IBM SPSS Statistics (Version 29.0, IBM Corp., Armonk, NY, USA).

We used the statistical program SAS 9.4 (SAS Institute Inc. 2013, Cary, NC, USA) to build a generalized linear regression model with a negative binomial distribution and a logit link using the studies from Europe (n = 16) or North Africa (n = 8). The year of sampling, region, and sample type, including the interaction between region and sample type, were used as independent variables, and the prevalence of ESBL-resistant *E. coli* (number of resistant samples out of all samples) was the dependent variable. The level of significance was set to 0.05. Incidence risk ratios (IRRs) were also calculated, and model diagnostics included visual inspection of residuals for normality and homoscedasticity. Cook’s distance and leverage were used to investigate outliers. To illustrate the interaction between region and sample type, a figure displaying the estimates and 95% confidence intervals (95% CI) was created using MS Excel^®^ (Microsoft Corporation, Redmond, WA, USA). North America was excluded because only three studies fulfilled the criteria.

### 4.8. Meta-Analyses

Since we assumed that the distribution of prevalences across the studies was heterogeneous, we used random-effects models to determine a pooled prevalence estimate, including a 95% CI using the Clopper–Pearson method for individual studies. The results are presented as forest plots displaying each study’s point estimates and confidence intervals, including summary and confidence intervals.

Heterogeneity was assessed by Cochran’s Q test and the inverse variance index I2. The I^2^ was 99.1% for feces and 98.2% for meat, indicating a very high level of heterogeneity in the studies (*p* < 0.0001 in both models). Due to the small number of studies included in the analyses, we limited our evaluation of heterogeneity to a Galbraith plot. Analyses and visualization were carried out using the meta package in R (version 4.3.1) [179].

### 4.9. Quality Assessment

Three people independently assessed quality (JK, RM, NS). We used the checklist for assessing the quality of quantitative studies based on the Standard Quality Assessment Criteria for evaluating primary research publications from a variety of fields from the Alberta Heritage Foundation for Medical Research [180].

Scores from 0 to 2 were given to each of the 11 questions concerning the sufficient description of the objectives; the appropriateness of the study design; the method and subject characteristics; whether the outcome and measures were well defined and robust to errors; the appropriateness of the samples size; whether analysis was described and appropriate; if estimates of variances in the main results were reported; if confounding was controlled; if the results were reported in a sufficient detail; and if the results supported the conclusion. The scores were summed to a total score ranging from 0 to 18. Articles with values of 8 to 11 were reassessed. The studies were divided into three groups depending on the total score (low risk of bias, medium risk of bias, and high risk of bias), and these were displayed using a bias plot in R. Publications with a score > 11 were regarded as high-quality and marked as low risk of bias in the bias plot. Publications were regarded as acceptable with scores of between 8 and 11 and shown as medium risk of bias, while those with scores of less than 8 were assessed as critical and displayed as high risk of bias in the bias plot. The studies grouped into these three categories (low, medium, or high risk of bias) were displayed in a bias plot generated with robvis (version 0.3.0; R Foundation for Statistical Computing, Vienna, Austria; DOI: 10.32614/CRAN.package.robvis). The approach addressed the quality of individual studies. No additional method, such as GRADE (Grading of Recommendations Assessment, Development and Evaluation), was applied to assess certainty in the body of evidence.

## Figures and Tables

**Table 1:** ESBL and AmpC resistance in fecal samples (50–100%, 9 studies).

Author	DOI	Year	Country	Bacteria	n Samples (Isolates)	% Pheno-ESBL	% Pheno-AmpC	% Geno-ESBL	% Geno-AmpC	Denominator
Mesa et al. [16]	10.1093/jac/dkl211	2006	Spain	*E. coli*	100 (51)	100%				S, I
Abreu et al. [18]	10.1089/fpd.2014.1796	2014	Spain	*E. coli*	260	91.15%		44.62%		S
De Koster et al. [19]	10.3390/antibiotics10080945	2021	Belgium, The Netherlands	*E. coli*	779	56.74%				S
Huijbers et al. [20]	10.1093/jac/dku178	2014	The Netherlands	*E. coli*	500 (500)	96.40%		61.36%	38.64%	I
Huijbers et al. [21]	10.1016/j.vetmic.2014.12.010	2014	The Netherlands	*E. coli*	150	86.67%		65.31%	34.69%	I
Smet et al. [22]	10.1128/AAC.01285-07	2008	Belgium	*E. coli*	489 (489)	60.33%	25.97%			S, I
Hering et al. [23]	10.1016/j.prevetmed.2016.01.003	2016	Germany	*E. coli*	295 (295)	77.63%				S
Sghaier et al. [24]	10.1016/j.jgar.2019.01.002	2019	Tunisia	*E. coli*, *Klebsiella* spp.	60 (60)	*E. coli* 56.70%, *Klebsiella* spp. 6.67%				I
Päivärinta et al. [25]	10.1111/zph.12277	2016	Finland	*E. coli*	495 (41)	97.56%	5.45%	31.71%	53.66%	I

Abbreviations: Pheno-ESBL/AmpC = phenotypic ESBL or AmpC resistance; Geno-ESBL/AmpC = genotypic ESBL or AmpC detection. Year = year of publication; Country = country where samples were collected; Denominator: S = sample-based prevalence (number of resistances per total number of samples); I = isolate-based prevalence (number of resistances per total number of isolates).

**Table 2:** ESBL and AmpC resistance in fecal samples (20–50%, five studies).

Author	DOI	Year	Country	Bacteria	n Samples (Isolates)	% Pheno-ESBL	% Geno-ESBL	Denominator
Costa et al. [26]	10.1016/j.vetmic.2009.03.029	2009	Portugal	*E. coli*	76 (152)	42.11%	91.18%	I
Geser et al. [27]	10.1186/1746-6148-8-21	2012	Switzerland	*E. coli*	93	43.30%		I
Fetahagić et al. [28]	10.2478/aiht-2021-72-3560	2021	Bosnia and Herzegovina	*E. coli*	108	36.00%		I
Balázs et al. [29]	10.1556/004.2021.00036	2021	Hungary	*E. coli*	114	34.21%	34.20%	S
Persoons et al. [30]	10.1089/mdr.2009.0062	2010	Belgium	*E. coli*	2249 (2076)	~38.00%		I

Abbreviations: Pheno-ESBL = phenotypic ESBL resistance; Geno-ESBL = genotypic ESBL detection. Year = year of publication; Country = country where samples were collected; Denominator: S = sample-based prevalence (number of resistances per total number of samples); I = isolate-based prevalence (number of resistances per total number of isolates).

**Table 3:** ESBL and AmpC resistance in fecal samples (<20%, five studies).

Author	DOI	Year	Country	Bacteria	n Samples (Isolates)	% Pheno-ESBL	% Pheno-AmpC	% Geno-ESBL	% Geno-AmpC	Denominator
Abbassi et al. [31]	10.1155/2021/1269849	2021	Tunisia	*E. coli*	170 (83)	1.2%		1.2%		I
Moawad et al. [32]	10.1186/s13099-018-0266-5	2018	Egypt	*E. coli*, KEC	576 (*E. coli* 56, KEC 9)	*E. coli* 12.50%, KEC 44.4%		20.00% ^a^	3.08% ^a^	I
Agunos et al. [33]		2018	Canada	*E. coli*	(271)	12.92%				I
Ceccarelli et al. [34]	10.1099/jmm.0.001176	2020	The Netherlands	*E. coli*	(1811)	6.18%				I
Wasyl et al. [35]	10.1089/mdr.2011.0033	2012	Poland	*E. coli*	181	4.70%	9.39%	0.55%	5.53%	S

Abbreviations: Pheno-ESBL/AmpC = phenotypic ESBL or AmpC resistance; Geno-ESBL/AmpC = genotypic ESBL or AmpC detection. Year = year of publication; Country = country where samples were collected; Denominator: S = sample-based prevalence (number of resistances per total number of samples); I = isolate-based prevalence (number of resistances per total number of isolates); KEC = *Klebsiella* spp., *Enterobacter* spp., and *Citrobacter* spp. ^a^ Combined prevalence for all listed *Enterobacteriaceae*.

**Table 10:** Comparison of phenotypic and genotypic fluoroquinolone resistance in six European studies.

Author	DOI	Year	Country	Sample Type	Bacteria	n Samples (Isolates)	% Pheno-R	% Geno-R	Denominator
Ghodousi et al. [63]	10.1089/fpd.2015.1936	2015	Italy	meat	*E. coli*	163 (134)	81.34%	81.34%	I
Röderova et al. [152]	10.3389/fmicb.2016.02147	2017	Czech Republic	bedding	*E. coli*	2628	5.93%	10.90%	S, I
Literak et al. [153]	10.1089/m d r.2012.0124	2013	Czech Republic	skin	*E. coli*	319 (114)	26.32%	4.30%	I
Kmet et al. [154]	10.1007/s12223-010-0013-x	2009	Slovakia	feces	*E. coli*	(317)	45.11%	0.32%	I
Kocúreková et al. [155]	10.3390/antibiotics10111303	2021	Slovakia	cloacal	*E. coli*	(115)	60.87%	n.r.	I
Racewicz et al. [156]	10.1038/s41598-022-09996-y	2022	Poland	litter, cloacal, neck skin	*E. coli*	(74)	litter 81.00%, feces 92.00%, meat 79.00%	31.00%	I

Abbreviations: Pheno/Geno-R = phenotypic/genotypic fluoroquinolone resistance; Year = year of publication; Country = country where samples were collected; n.r. = not reported (genotypically tested but no numerical data reported); Denominator: S = sample-based prevalence (number of resistances per total number of samples); I = isolate-based prevalence (number of resistances per total number of isolates).

**Table 11:** Comparison of phenotypic and genotypic fluoroquinolone resistance in seven North African studies.

Author	DOI	Year	Country	Sample Type	Bacteria	n Samples (Isolates)	% Pheno-R	% Geno-R	Denominator
Awad et al. [42]	10.1186/s12941-016-0174-9	2016	Egypt	organ	*E. coli*	400 (116)	41.38%	33.33%	I
Laarem et al. [157]	10.3855/jidc.8643	2016	Algeria	meat	*E. coli*	33	45.45%	9.09%	S
Messaili et al. [53]	10.12834/VetIt.799.3865.2	2019	Algeria	intestine	*E. coli*	100 (100)	62.00%	13.00%	I
Mnif et al. [123]	10.1111/j.1472-765X.2012.03309.x	2012	Tunisia	feces	*E. coli*	136	71.6% of ESBL positives	8.96%	S
Moawad et al. [76]	10.1186/s13099-017-0206-9	2017	Egypt	meat	*E. coli*	90 (15)	26.67%	33.33%	I
Belmahdi et al. [55]	10.1016/j.jgar.2016.04.006	2016	Algeria	cecal	*E. coli*	61 (61)	90.00%	0.00%	I
Agabou et al. [126]	10.1007/s10096-015-2534-3	2015	Algeria	feces	*E. coli*	70	51.42%	11.43%	S

Abbreviations: Pheno/Geno-R = phenotypic/genotypic fluoroquinolone resistance; Year = year of publication; Country = country where samples were collected; Denominator: S = sample-based prevalence (number of resistances per total number of samples); I = isolate-based prevalence (number of resistances per total number of isolates).

**Table 12:** Results of a regression model with a negative binomial distribution, investigating the influence of region, year, and sample type on the prevalence of ESBL-*E. coli* in meat or feces samples; 24 studies (16 from Europe, 8 from North Africa, 11 on feces, 13 on meat).

Parameter	Estimate	Standard Error	95% CI	*p*-Value
Intercept	135.7128	85.9145	−32.6765–304.1021	0.1142
Region (Europe)	1.6908	0.4380	0.8324–2.5492	0.0001
Region (North Africa)	0.0000	0.0000	0.0000–0.0000	
Year	−0.0686	0.0426	−0.1522–0.0149	0.1228
Sample type (Feces)	1.3435	0.4514	0.4587–2.2283	0.0029
Sample type (Meat)	0.0000	0.0000	0.0000–0.0000	
Region (Europe) × Sample type (Feces)	−1.7540	0.5319	−2.7965–(−0.7115)	0.0010
Region (Europe) × Sample type (Meat)	0.0000	0.0000	0.0000–0.0000	
Region (North Africa) × Sample type (Feces)	0.0000	0.0000	0.0000–0.0000	
Region (North Africa) × Sample type (Meat)	0.0000	0.0000	0.0000–0.0000	
Distribution	0.2873	0.0830	0.1631–0.5061	

**Table 13:** Inclusion criteria for study selection.

Population	Broiler Chicken
Sample categories	Fecal (feces, cloacal swabs). Meat (retail meat, intended for consumption, collected at slaughterhouses). Environmental (bedding, dust, litter, cage swabs, wastewater, air, or flies within barns). Other than feces (cecal samples, boot swabs, organ samples).
Bacteria	*E. coli*, *Klebsiella* spp., *Enterobacter* spp., and *Citrobacter* spp.
Resistance patterns	Beta-Lactam Resistance (ESBLs, AmpC) Carbapenemases Colistin Resistance Fluoroquinolones Resistance
Region	Europe North Africa (Algeria, Egypt, Tunisia, Morocco) North America (USA, Canada)
Language	English or German
Time	1 January 2002–31 July 2022

## Data Availability

The original contributions presented in this study are included in this article/Supplementary Materials. Further inquiries can be directed to the corresponding authors.

## Supplementary Materials

The following supporting information can be downloaded at https://www.mdpi.com/2079-6382/14/12/1268/s1: Table S1: Beta-lactam; Table S2: 25 Sorted-out beta-lactam; Table S3: Carbapenemases; Table S4: Colistin; Table S5: Fluoroquinolone; Table S6: Figure 3, Genotypic R per isolates.
