# Supplementary material for: Antimicrobial Resistance in Selected Enterobacteriaceae from Broilers and Their Environment: ESBL, AmpC, Carbapenemases, Colistin, and Fluoroquinolone Resistance—A Systematic Review and Meta-Analysis
Source: Antibiotics (Basel). 2025 Dec 15;14(12):1268. doi: 10.3390/antibiotics14121268 (PMC12865486; doi:10.3390/antibiotics14121268)
Supplement: Supplementary file 1 [file antibiotics-14-01268-s001.zip › antibiotics-3970136-supplementary/Supplemantary_Table_S5_Fluoroquinolone.pdf]

Table S5. Studies of Fluoroquinolone resistance (92)

Author	DOI	Year	Country	Sample type	Bacteria	n (samples)	n (isolates)	Pheno-R (n)	Pheno-R (S)	Pheno-R (I)	Geno-R (n)	Geno-R (S)	Geno- R (I)
Abbassi et. al	10.1155/2021/1269849	2021	Tunisia	fecal samples	*E. coli*	170	83	27	15.88%	32.50%			
Abreu et. al	10.1089/fpd.2014.1796	2014	Spain	cloacal samples	*E. coli, Klebsiella spp.*	260		155	65.4 %				
Awad et. al	10.1186/s12941-016-0174-9	2016	Egypt	organ samples	*E. coli*	400	116	48	12.00%	41.38%	16		33.33%
Badr et. al	10.3390/ani12030346	2022	Egypt	organ samples	*E. coli*	120	56	37	30.83%	66.07%			
Balázs et. al	10.1556/004.2021.00036	2021	Hungary	fecal samples	*E. coli*	114		39		100%			
Benameur et. al	10.3390/antibiotics10101157	2021	Algeria	fecal samples	*E. coli*	32	18	16		89% of 18 strains			
Hassen et. al	10.1016/j.ijfoodmicro.2019.108478	2019	Tunisia	fecal samples + meat samples	*E. coli*	333	333	81		86,1%			
Belmar Campos et. al	10.1016/j.ijmm.2014.04.012	2014	Germany	meat samples	*E. coli, Klebsiella spp.*	120	87	20	23%				
Casella et. al	10.1016/j.ijfoodmicro.2017.07.005	2017	France	meat samples	*E. coli*	48	77	16		20.78%			
Clemente et. al	10.3390/antibiotics10111333	2021	Portugal	meat samples	*E. coli*	198	60	56	28.28%	93.30%	23	11.62%	38.33%
Cohen Stuart et. al	10.1016/j.ijfoodmicro.2011.12.034	2012	Netherlands	meat samples	*E. coli, Klebsiella spp.*	98	163	13		14%			

Costa et. al	10.1016/j.vetmic.2009.03.029	2009	Portugal	fecal samples	*E. coli*	76	152	18		56.25%			
Ghodousi et. al	10.1089/fpd.2015.1936	2015	Italy	meat samples	*E. coli*	163	134	109	66.87%	81.34%	109		81.34%
Dhaouadi et. al	10.1016/j.jgar.2020.03.017	2020	Tunisia	organ samples	*E. coli*	100	50	34	34.00%	68.00%			
Dierikx et. al	10.1016/j.vetmic.2010.03.019	2010	Netherlands	caecal samples	*E. coli*		153	77		50.00%			
Egea et. al	10.1016/j.ijfoodmicro.2012.08.002	2012	Spain	meat samples	*E. coli*	15		20		32.26%, but Broiler and Turkey mixed			
Elmonir et. al	10.3390/biology10050373	2021	Egypt	organ samples + litter + water + food samples	*Klebsiella spp.*	160	19	14 Klebsiella spp.		72.90%			
El-Shazly et. al	10.3382/ps/pew493	2017	Egypt	cloacal samples	*E. coli*		50	41		82.00%			
Fetahagić et. al	10.2478/aiht-2021-72-3560	2021	Bosnia and Herzegovina	fecal samples	*E. coli*	108		8		10.67%			
Geser et. al	10.1186/1746-6148-8-21	2012	Switzerland	fecal samples	*E. coli*	93		4		6.45%			
Gregova et. al		2012	Slovakia	samples from processing plant of a slaughterhouse	*E. coli*		48	21		43.00%			
Hadžić-Hasanović et. al	10.17392/1206-20	2020	Bosnia and Herze	chicken skin	*E. coli*	100	64	26		89.65%			

			govina										
Homeier-Bachmann et. al	10.3390/antibiotics10050568	2021	Germany	wastewater (slaughterhouse)	*E. coli, Enterobacter spp., Klebsiella spp.*		26	29		53.00%			
Hricová et al.	10.21101/cejph.a4328	2017	Czech Republic	bedding (boot swabs)	*E. coli*	126	126	77	61.11%	61.11%			
Blanc et. al	10.1016/j.vetmic.2006.08.002	2006	Spain	fecal samples	*E. coli*		192	20	10.42%	31.20%			
Laarem et al.	10.3855/jidc.8643	2016	Algeria	meat samples	*E. coli*	33		15	45.45%	51.72%	3	9.09%	10.34%
Kola et. al	10.1093/jac/dks295	2012	Germany	meat samples	*E. coli, Enterobacter spp.*	399		14		7,6%			
Machado et. al	10.1093/jac/dkn179	2008	Portugal	carcasses (meat) + fecal samples		40	101	5 E. coli, 2 Klebsiella spp.		50% *E. coli*, 50% *Klebsiella* spp.			
Martínez-Álvarez et al.	10.3390/antibiotics11040444	2022	Spain	Air + Manure	*E. coli*	111	111	43	38.70%	38.70%			
Messaili et al.	10.12834/VetIt.799.3865.2	2019	Algeria	intestine	*E. coli*	100	100	62	62.00%	62.00%	13		13.00%
Michael et. al	10.1016/j.vetmic.2016.08.023	2016	Germany	fecal samples + intestine	*E. coli*		2391	4		20%			
Mnif et. al	10.1111/j.1472-765X.2012.03309.x	2012	Tunisia	fecal samples	*E. coli*	136		51	71,6%		6	8.96%	
Moawad et al.	10.1186/s13099-017-0206-9	2017	Egypt	meat samples	*E. coli*	90	15	4	4.44%	26.67%	5	5.56%	33.33%

Moawad et al.	10.1186/s13099-018-0266-5	2018	Egypt	cloacal samples	*E. coli, Enterobacter spp., Klebsiella spp., Citrobacter spp.*	576	65	12 (1 *Enterobacter* spp., 2 *Klebsiella* spp., 0 *Citrobacter* spp.)	2.08% (0% *Enterobacter/Klebsiella/Citrobacter* spp.)	21.43% (20% *Enterobacter* spp., 100% *Klebsiella* spp., 0% *Citrobacter* spp.)	6	1.04%	9.23%
Musa et. al	10.3390/ani10071215	2020	Italy	cloacal samples + skin samples	*E. coli*		406	120		29,56% total, 20.6% ABF, 23,6% O, 44,4 C			
Röderova et al.	10.3389/fmicb.2016.02147	2017	Czech Republic	bedding (boot swabs)	*E. coli*	2628		156	5.94%	5.94%	17	10.90%	10.90%
Ramadan et. al	PMC5715280	2017	Egypt	meat samples	*Klebsiella spp.*	360	80	32		40.00%			
Ramadan et. al	10.3390/pathogens9050357	2020	Egypt	carcasses (meat)	*E. coli*	61		52		43.30%			
Sheikh et. al	10.1089/fpd.2011.1078	2012	Canda	meat samples	*E. coli*	206	193	0	0.00%	0.00%			
Smet et. al	10.1128/AAC.01285-07	2008	Belgium	cloacal samples	*E. coli*	489	489	25	5.11%	8.47%			
Zarfel et. al	10.3390/ijerph111212582	2014	Austria	meat samples	*E. coli*	50		0	0.00%				
De Koster et al.	10.3390/antibiotics10080945	2021	Belgium, Nethe rlands	fecal samples	*E. coli*	779		497	84.81%				
Belmahdi et al .	10.1016/j.jgar.2016.04.006	2016	Algeria	caecal samples	*E. coli*	61	61	18	29.51%	90.00%		0.00%	0.00%
Awosile et. al	10.1139/cjm-2020-0442	2020	Canada	meat samples	*E. coli*	144		3	2.08%				

García-Béjar et. al	10.3390/ani11113197	2021	Spain	meat samples	*E. coli*	30	240			ca. 25%			
Kmet et al.	10.1007/s12223-010-0013-x	2009	Slovakia	fecal samples	*E. coli*		317	143		45.11%	1	16.67%	0.32%
Kocúreková et al.	10.3390/antibiotics10111303	2021	Slovakia	cloacal samples	*E. coli*		115	70		60.87%			
Randall et. al	10.1093/jac/dkq396	2010	UK	caecal samples	*E. coli*	388		18		4.60%			
Manageiro et. al	10.1016/j.ijfoodmicro.2017.10.007	2017	Portugal	caecal samples	*E. coli*	680	202	183	90.60%	90.60%	1 but unclear (broiler or turkey)		
Much et. al	10.1016/j.prevetmed.2019.104755	2019	Austria	caecal samples	*E. coli*	1031	962	614		63.83%			
Niero et al.	10.1016/j.vetmic.2018.02.012	2018	Italy	organ samples	*E. coli*	98	98				4	4.08%	4.08%
Pesciaroli et. al	10.1016/j.ijfoodmicro.2019.108391	2019	Italy	caecal samples	*E. coli*	855	854	444	51.99%	52% (67.7% in conventional samples, 42.8% in antibiotic-free samples and 45.2% in organic samples)			
Sabate et. al	10.1016/j.resmic.2008.02.001	2008	Spain	wastewater (slaughterhouse)	*E. coli*	4	43	24		56.00%			
Savin et al .	10.1016/j.scitotenv.2021.150000	2021	Germany	water samples (slaughterhouse) + area	*Klebsiella spp.*	82	71				3	3.66%	4.23%

				of slaughterhouses									
Chenouf et. al	10.1089/mdr.2020.0024	2020	Algeria	retail liver	*E. coli, Klebsiella spp.*	136	78	44	9.40%	60.20%			
Literak et al .	10.1089/m d r.2012.0124	2013	Czech Republic	skin samples	*E. coli*	319	114	30		26.32%	4	1.25%	4.30%
Maciuca et. al	10.1089/mdr.2014.0248	2015	Romania	caecal samples	*E. coli*	127	90	118		87.70%			
Saidani et. al	10.1089/mdr.2019.0138	2019	Tunisia	cloacal samples	*E. coli, Klebsiella spp.*	258		34		86%			
Schwaiger et. al	10.1089/mdr.2012.0257	2013	Germany	fecal samples	*E. coli*		438	4		0.91%			
Vogt et. al	10.1089/mdr.2013.0210	2014	Switzerland	meat samples	*E. coli*	75	68	1		1,45%			
Vounba et. al	10.1089/mdr.2018.0403	2019	Canada	fecal samples	*E. coli*			1		1.01%			
Mesa-Varona et al.	10.1371/journal.pone.0243772	2020	Germany	caecal samples	*E. coli*		592	426	51.43%	71.96% total; 61,6% clinical; 53% non-clinical, CI=1,30 (0,98-1,73); p=0,064)			
Agabou et al.	10.1007/s10096-015-2534-3	2015	Algeria	fecal samples	*E. coli*	70		36		51.43%	8	11.43%	22.20%
Amer et. al	10.14202/vetworld.2018.1082-1088	2018	Egypt	organ samples	*E. coli*	160	56	42		75.00%			

Agunos et al.		2018	Canada	cloacal/ fecal samples	*E. coli*		271	0		0.00%			
Amato et al.	10.1016/j.scitotenv.2020.139401	2020	USA	waterways near confined poultry operations	*E. coli*		337	0		0.00%			
Bessalah et al.	10.1080/10495398.2020.1752702	2020	Tunisia	fecal samples	*E. coli*	17	7	0		0.00%			
Boulianne et al.		2015	Canada	caecal samples	*E. coli*	1500	962	0		0.00%			
Ceccarelli et al .	10.1099/jmm.0.001176	2020	Netherlands	fecal samples	*E. coli*		1811	1127		62.23%			
Gousia et al.	10.1089=fpd.2010.0577	2011	Greece	meat samples	*E. coli*	19	8	5		62.50%			
Hamed et al.	10.1155/2021/6739220	2021	Egypt	organ samples	*E. coli*	657	496	352		71.00%			
Hanon et al.	10.1016/j.prevetmed.2015.09.001	2015	Belgium	caecal samples	*E. coli*	1132	1132						
Huang et. al	10.1637/8268-021608-Reg.1	2008	USA	organ samples	*E. coli*		445	15		3.37%			
Kaesbohrer et al.	10.1111/j.1863-2378.2011.01451.x	2012	Germany	fecal samples + meat samples	*E. coli*		397	87 bzw 103		meat: 103/194, 53,1%; feces: 87/202; 43,1%			
Mainali et al.	10.4315/0362-028X.JFP-13-203	2013	Canada	caecal samples	*E. coli*			0		0.00%			
Mohamed et. al	10.1155/2014/195189	2014	Egypt	organ samples + cloacal swabs	*E. coli*	303	25	25		100.00%			
Musa et al.	10.3390/antibiotic	2021	Italy	cloacal samples, ceacal	*E. coli*		406	34 (only conventional)		A-free 41,4%; organic			

	s10111321			samples, skin samples						39,7%; conventional 58,6%			
Myrenås et al.	10.1016/j.vetmic.2017.11.015	2017	Norway, Sweden, Iceland	ceacal samples + meat samples (not distinguishable)	*E. coli*		319 (not distinguishable between caecal and meat samples)	43		13,5% (unclear if caecal or meat samples)			
Pasquali et al.	10.1016/j.vetmic.2015.05.007	2015	Italy	organ samples	*E. coli*	289	322	118		36,65%			
Pavlickova et al.	10.1080/03601234.2015.1011959	2014	Czech Republic	meat samples	*E. coli*		75	12		16.00%			
Persoons et. al	10.1089/mdr.2009.0062	2010	Belgium	cloacal samples, caecal samples, neck skin	*E. coli, Enterobacter spp.*	2249	2076	108		14.98%			
Racewicz et al.	10.1038/s41598-022-09996-y	2022	Poland	litter samples + cloacal samples + neck skin	*E. coli*		74			81% litter, 92% fecal samples, 79% meat			31.08%
Romero-Barrios et al.	10.1089/fpd.2019.2776	2020	Canada	carcasses	*E. coli*		1135	2		0.18%			
Ružauskas et al.		2010	Lithuania	chicken liver (retail)	*E. coli*	240	100	47		47.00%			
Samy, A. A et al.	10.14202/vetworld.2022.488-495	2022	Egypt	Liver, intestinal content, blood, and bone marrow	*E. coli*	350	132	67		50,8%			

Savin et. al	10.3390/antibiotics11040435	2022	Germany	slaughterhouse wasterwater (poultry and pig; keine Unterscheidung in den Ergebnissen)	*Klebsiella spp.*								
Sgarigli a et al.	10.12834/VetIt.1617.8701.1	2019	Italy	liver + intestinal swab (Zahlen nicht nutzbar)	*E. coli*	Different animal species, not distinguishable	Different animal species, not distinguishable						
Thorsteinsdottir et al.	10.1111/j.1863-2378.2009.01256.x	2008	Iceland	ceacal samples + meat samples	*E. coli*	146	185			18,2% (20/110 cecal) 36% (27/75 meat)			
Vanni, M. et al.	10.3382/ps.2013-03627	2014	Italy	fecal samples + organ samples	*E. coli*		235			24,2% (57/235 ; unclear: mixed fecal and organ samples)			
Varga et. al	10.1186/s12917-019-2187-z	2019	Canada	ceacal samples	*E. coli*		358	1	72.24%	0,28% (1/358)			
Wasyl et. al	10.3389/fmicb.2013.00221	2013	Poland	cloacal samples	*E. coli*	753	682	544		79.77%			
Zhao et al.	10.1128/AEM.07522-11	2012	USA	meat samples	*E. coli*		2494	0		0% (0/2494)			

Abbreviations: n = number; Pheno-R = phenotypic resistance; Geno-R = genotypic resistance; S = per samples; I = per isolates; Year = year of publication; Country = country where samples were collected
