# Supplementary material for: Antimicrobial Resistance in Selected Enterobacteriaceae from Broilers and Their Environment: ESBL, AmpC, Carbapenemases, Colistin, and Fluoroquinolone Resistance—A Systematic Review and Meta-Analysis
Source: Antibiotics (Basel). 2025 Dec 15;14(12):1268. doi: 10.3390/antibiotics14121268 (PMC12865486; doi:10.3390/antibiotics14121268)
Supplement: Supplementary file 1 [file antibiotics-14-01268-s001.zip › antibiotics-3970136-supplementary/Supplementary_Table_S2_25_sorted out_Beta_Lactam.pdf]

Table S2. Studies mentioning Beta-Lactam resistance but excluded from data analysis (25)

**Author**	**DOI**	**Year**	**Country**	**Sample type**	**Bacteria**	**n (samples)**	**n (isolates)**	**Pheno-R ESBL (n)**	**Pheno-R ESBL (S)**	**Pheno-R ESBL (I)**	**Geno-R ESBL (n)**	**Geno-R ESBL (S)**	**Geno-R ESBL (I)**	**Pheno-R AmpC (n)**	**Pheno-R AmpC (S)**	**Pheno-R AmpC (I)**	**Geno-R AmpC (n)**	**Geno-R AmpC (S)**	**Geno-R AmpC (I)**
Agabou et al.	10.1007/s10096-015-2534-3	2015	Algeria	fecal	*E. coli*	70	70.0	0	0.00%	0.00%	8								
Amato et al.	10.1016/j.scitotenv.2020.139401	2020	USA	waterways near confined poultry operations	*E. coli*		337.0	21		6.00%									
Ben Sallem et. al	10.1089/fpd.2012.1267	2012	Tunisia	fecal	*E. coli*	22 (bad quality)		10	45.45%	45.45%	8	36.36%		2	9.09%	9.09%	2	9.09%	
Benameur et. al	10.3390/antibiotics10101157	2021	Algeria	fecal	*E. coli*	32	18.0	10	31.25%	55.56%	10	55.56%	55.56%						
Bessalah et al.	10.1080/10495398.2020.1752702	2020	Tunisia	fecal	*E. coli*	17 (bad quality)	7.0	0	0.00%	0.00%									
Dahms et. al	10.1371/journal.pone.0143326	2015	Germany	cloacal	*E. coli*					No data at sample or isolate level; 3 out of 4 farms positive	3								

Egea et. al	10.1016/j.ijfoodmicro.2012.08.002	2012	Spain	meat	*E. coli*	15		39	93.33%										
Galler et. al	10.3390/antibiotics10040466	2021	Austria	intestine	*E. coli*	100 (bad quality)		16			16		45.71%						
Gousia et al.	10.1089=fpd.2010.0577	2011	Greece	meat	*E. coli*	19	8.0	3		37,5% (cefurixime, 0% ceftriaxone)									
Halfaoui et. al	10.14202/vetworld.2017.830-835	2017	Algeria	organ	*E. coli*	180 (bad quality)	156.0	3	1.68%	1.94%	3	1.92%	1.92%						
Kilani et. al	10.3389/fcimb.2015.00038	2015	Tunisia	fecal	*E. coli*	65 (bad quality)	65.0	17	26.15%	26.15%	16	24.62%	24.62%						
Kmet et al.	10.1007/s12223-010-0013-x	2009	Slovakia	fecal	*E. coli*	bad quality	317.0				0		0.00%						
Kocúreková et al.	10.3390/antibiotics11011303	2021	Slovakia	cloacal	*E. coli*		115.0	11		9.48%	2		1.74%						
Leverstein-van Hall et. al	10.1111/j.1469-0691.2011.03497.x	2011	Netherlands	meat + ceacal	*E. coli*	bad quality	116.0				116		100.00%						
Liebana et. al	10.1089/1076629043230477 45	2004	UK	environmental from chicken farms	*E. coli*		2.0				1		50.00%	2		100.00%			

Machado et. al	10.1093/jac/dkn179	2008	Portugal	carcasses (meat) + fecal	*E. coli, Klebsiella spp.*	40 (bad quality)		12 E. coli, 4 Klebsiella spp.	30% E. coli, 3.96 % Klebsiella spp.	11.88 %	16	15.84%	15.84%						
Mnif et. al	10.1111/j.1472-765X.2012.03309.x	2012	Tunisia	fecal	*E. coli*	136 (bad quality)		43	31.62%		43	31.62%		224	164.71%		24	17.65 %	
Mohamed et. al	10.1155/2014/195189	2014	Egypt	organ + cloacal swabs	*E. coli*	303 (bad quality)	25.0	19		76.00 %									
Poudel et. al	10.3390/ijerph16193583	2019	USA	flies	*E. coli, Klebsiella spp.*	40 (bad quality)	31.0	0 E. coli und Klebsiella spp.	0.00 %	0.00%	0	0.00%	0.00%	0 E. coli und Klebsiella spp.	0.00 %	0.00 %	0	0.00%	0.00%
Ramadan et. al	10.1089/vbz.2017.2237	2018	Egypt	caecal + organ	*E. coli*	160	50.0	1	0.62 %	2.00%	16	32.00%	32.00%				17	10.62 %	34.00 %
Rojs et. al	10.3382/japr/pfy021	2019	Slovenia	Meconium, Air, organs, boot swabs	*E. coli*		116.0	37	31.90%	31.90 %									
Sadek et. al	10.3390/microorganisms9010195	2021	Egypt	neck skin	*E. coli, Citrobacter spp.*	345	20.0	9	2.61 %	45.00 %	17	4.93%							
Sgariglia et al.	10.12834/VetIt.1617.8701.1	2019	Italy	liver + intestinal swab	*E. coli*	Numbers cannot be extracted, different animal													

						species, bad quality													
Smith et. al	10.1128/AEM.01193-06	2007	USA	fecal	*E. coli*		450.0												
Vounba et. al	10.1089/mdr.2018.0403	2019	Canada	fecal	*E. coli*			108	90.70%		3						54	No calculation possible	

Abbreviations: n = number; Pheno-R = phenotypic resistance; Geno-R = genotypic resistance; S = per samples; I = per isolates; Year = year of publication; Country = country where samples were collected
