# Supplementary material for: Antimicrobial Resistance in Selected Enterobacteriaceae from Broilers and Their Environment: ESBL, AmpC, Carbapenemases, Colistin, and Fluoroquinolone Resistance—A Systematic Review and Meta-Analysis
Source: Antibiotics (Basel). 2025 Dec 15;14(12):1268. doi: 10.3390/antibiotics14121268 (PMC12865486; doi:10.3390/antibiotics14121268)
Supplement: Supplementary file 1 [file antibiotics-14-01268-s001.zip › antibiotics-3970136-supplementary/Supplementary_Table_S3_ Carbapenemases.pdf]

## *Table S3. Studies of carbapenem resistance (54)*

| Author | DOI | Year | Country | Sample type | Bacteria | n (samples) | n (isolates) | Pheno-R (n) | Pheno-R (S) | Pheno-R (I) | Geno-R (n) | Geno-R (S) | Geno-R (I) |
|---|---|---|---|---|---|---|---|---|---|---|---|---|---|
| Abbassi et. al | 10.1155/2021/1269849 | 2021 | Tunisia | fecal samples | *E. coli* | 170 | 83 | 0 | 0.00% | 0.00% | | | |
| Abdallah et. al | 10.1371/journal.pone.0136052 | 2015 | Egypt | carcasses (meat) | *E. coli, Enterobacter spp., Klebsiella spp., Citrobacter spp.* | 100 | 106 | | 10.7% | 26.1% | 12 | 12.00% | 11.32% |
| Abreu et. al | 10.1089/fpd.2014.1796 | 2014 | Spain | cloacal samples | *E. coli* | 260 | | 0 | 0.00% | 0.00% | | | |
| Agabou et al. | 10.1007/s10096-015-2534-3 | 2015 | Algeria | fecal samples | *E. coli* | 70 | 56 | 0 | 0.00% | 0.00% | | | |
| Amato et al. | 10.1016/j.scitotenv.2020.139401 | 2020 | USA | waterways near confined poultry operations | *E. coli* | | | 0 | 0.00% | 0.00% | | | |
| Badr et. al | 10.3390/ani12030346 | 2022 | Egypt | organ samples | *E. coli* | 120 | 333 | 0 | 0.00% | 0.00% | 10 | 8.33% | 17.86% |
| Balázs et. al | 10.1556/004.2021.00036 | 2021 | Hungary | fecal samples | *E. coli* | 114 | 87 | 0 | 0.00% | 0.00% | | | |
| Belmahdi et al . | 10.1016/j.jgar.2016.04.006 | 2016 | Algeria | caecal samples | *E. coli* | 61 | 77 | 0 | 0.00% | 0.00% | | | |

| | | | | | | | | | | | | | |
|---|---|---|---|---|---|---|---|---|---|---|---|---|---|
| Belmar Campos et. al | 10.1016/j.ijmm.2014.04.012 | 2014 | Germany | meat samples | *E.coli, Klebsiella spp.* | 120 | | 0 | 0.00% | 0.00% | | | |
| Casella et. al | 10.1016/j.ijfoodmicro.2017.07.005 | 2017 | France | meat samples | *E. coli* | 48 | 60 | 0 | 0.00% | 0.00% | | | |
| Chenouf et. al | 10.1089/mdr.2020.0024 | 2020 | Algeria | retail liver | *E. coli, Klebsiella spp.* | 136 | 152 | 0 | 0.00% | 0.00% | | | |
| Clemente et. al | 10.3390/antibiotics10111333 | 2021 | Portugal | meat samples | *E. coli* | 198 | 134 | 0 | 0.00% | 0.00% | | | |
| Chaalal et. al | 10.1089/mdr.2019.0419 | 2019 | Algeria | meat samples | *Klebsiella spp.* | 181 | | | 16.0% | | 29 | 16.02% | |
| Costa et. al | 10.1016/j.vetmic.2009.03.029 | 2009 | Portugal | fecal samples | *E. coli* | 76 | 19 | 0 | 0.00% | 0.00% | | | |
| De Koster et al. | 10.3390/antibiotics10080945 | 2021 | Belgium, Netherlands | fecal samples | *E. coli* | 779 | 50 | 0 | 0.00% | 0.00% | | | |
| Egea et. al | 10.1016/j.ijfoodmicro.2012.08.002 | 2012 | Spain | meat samples | *E. coli* | 15 | | 0 | 0.00% | 0.00% | | | |
| El-Shazly et. al | 10.3382/ps/pew493 | 2017 | Egypt | cloacal samples | *E. coli* | | | 0 | | 0.00% | | | |
| Facciolà et. al | 10.3390/ijerph18189611 | 2021 | Italy | wastewater (slaughterhouse) | *E. coli, Citrobacter spp.* | 4 | 64 | 0 | 0.00% | 0.00% | | | |
| Fetahagić et. al | 10.2478/aiht- | 2021 | Bosnia and | fecal samples | *E. coli* | 108 | 26 | 0 | 3.1% | 26.3% | | | |

| | | | | | | | | | | | | | |
|---|---|---|---|---|---|---|---|---|---|---|---|---|---|
| | 2021-72-3560 | | Herzegovina | | | | | | | | | | |
| Elmonir et. al | 10.3390/biology10050373 | 2021 | Egypt | organ samples + litter + water + food samples | *Klebsiella spp.* | 160 | 2391 | | | ca. 70% | 4 | 2.50% | 21.05% |
| García-Béjar et. al | 10.3390/ani11113197 | 2021 | Spain | meat samples | *E. coli* | 30 | | | | 0.00% | | | |
| Geser et. al | 10.1186/1746-6148-8-21 | 2012 | Switzerland | fecal samples | *E. coli* | 93 | 15 | 0 | 0.00% | 0 .00% | | | |
| Ghodousi et. al | 10.1089/fpd.2015.1936 | 2015 | Italy | meat samples | *E. coli* | 163 | 65 | 0 | | 0.00% | | | |
| Gousia et al. | 10.1089=fpd.2010.0577 | 2011 | Greece | meat samples | *E. coli* | 19 | | 0 | 0.00% | 0.00% | | | |
| Hadžić-Hasanović et. al | 10.17392/1206-20 | 2020 | Bosnia and Herzegovina | chicken skin | *E. coli* | 100 | | 0 | 0.00% | 0.00% | | | |
| Hassen et. al | 10.1016/j.ijfoodmicro.2019.108478 | 2019 | Tunisia | fecal samples + meat samples | *E. coli* | 333 | 50 | 0 | 0.00% | 0.00% | | | |
| Hamza et. al | 10.1016/j.jgar.2016.06.004 | 2016 | Egypt | organ samples + fecal samples | *Klebsiella spp.* | 100 | 183 | 43 | 15% | 43% | 15 | 15.00% | 42.86% |
| Homeier-Bachmann et. al | 10.3390/antibiotics10050568 | 2021 | Germany | wastewater (slaughterhouse) | *E. coli, Enterobacter spp., Klebsiella spp.* | | | 0 | | 0.00% | | | |
| Kocúreková et al. | 10.3390/antibiotics1 | 2021 | Slovakia | cloacal samples | *E. coli* | | | 0 | | 0.00% | | | |

| | | | | | | | | | | | | | |
|---|---|---|---|---|---|---|---|---|---|---|---|---|---|
| | 0111303 | | | | | | | | | | | | |
| Maciuca et. al | 10.1089/mdr.2014.0248 | 2015 | Romania | caecal samples | *E. coli* | 127 | 61 | 0 | | 0.00% | | | |
| Manageiro et. al | 10.1016/j.ijfoodmicro.2017.10.007 | 2017 | Portugal | caecal samples | *E. coli* | 680 | 35 | 0 | 0.00% | 0.00% | | | |
| Michael et. al | 10.1016/j.vetmic.2016.08.023 | 2016 | Germany | fecal samples + intestine | *E. coli* | | 240 | 0 | | 0.00% | | | |
| Mnif et. al | 10.1111/j.1472-765X.2012.03309.x | 2012 | Tunisia | fecal samples | *E. coli* | 136 | 115 | 0 | 0.00% | | | | |
| Moawad et al. | 10.1186/s13099-017-0206-9 | 2017 | Egypt | meat samples | *E. coli* | 90 | 202 | | | 0.00% | 3 | 3.33% | 20.00% |
| Moawad et al. | 10.1186/s13099-018-0266-5 | 2018 | Egypt | cloacal samples | *E. coli, Enterobacter spp., Klebsiella spp., Citrobacter spp.* | 576 | 962 | 0.017857142857142856 | 0.00% | 0.00% | | | |
| Mollenkopf et. al | 10.1089/fpd.2017.2390 | 2018 | USA | cage swab samples, retail ground chicken | *E. coli* | 497 | 605 | 0 | 0.00% | | | | |
| Much et. al | 10.1016/j.prevetmed.2019.104755 | 2019 | Austria | caecal samples | *E. coli* | 1031 | 854 | 0 | 0.00% | 0.00% | | | |
| Musa et al. | 10.3390/antib | 2021 | Italy | cloacal samples, | *E. coli* | | | 0 | 0.00% | 0.00% | | | |

| | | | | | | | | | | | | | |
|---|---|---|---|---|---|---|---|---|---|---|---|---|---|
| | iotics10111321 | | | ceacal samples, skin samples | | | | | | | | | |
| Ojer-Usoz et. al | 10.1016/j.meatsci.2012.09.009 | 2012 | Spain | meat samples | *E. coli, Enterobacter spp., Klebsiella spp.* | | 71 | 0 | 0.00% | | | | |
| Päivärinta et. al | 10.1016/j.ijfoodmicro.2019.108361 | 2019 | Finland | caecal samples + meat samples | *E. coli* | 605 | 60 | | | 0.00% | 0 | 0.00% | 0.00% |
| Pavlickova et al. | 10.1080/03601234.2015.1011959 | 2014 | Czech Republic | meat samples | *E. coli* | | 78 | 0 | 0.00% | 0.00% | | | |
| Pesciaroli et. al | 10.1016/j.ijfoodmicro.2019.108391 | 2019 | Italy | caecal samples | *E. coli* | 855 | 90 | 0 | 0.00% | | | | |
| Ramadan et. al | 10.1089/vbz.2017.2237 | 2018 | Egypt | caecal samples + organ samples | *E. coli* | 160 | | | | 0.00% | | | |
| Ramadan et. al | 10.3390/pathogens9050357 | 2020 | Egypt | carcasses (meat) | *E. coli* | 61 | 68 | 0 | 0.00% | 0.00% | 0 | 0.00% | 0.00% |
| Randall et. al | 10.1111/jam.14687 | 2020 | UK | meat samples | *E. coli* | 622 | | 0 | 0.00% | 0.00% | | | |
| Randall et. al | 10.1016/j.ijfoodmicro.2016.10.036 | 2016 | UK | meat samples | *E. coli* | 159 | 337 | 0 | 0.00% | 0.00% | | | |
| Saidani et. al | 10.1089/mdr.2019.0138 | 2019 | Tunisia | cloacal samples | *E. coli, Klebsiella spp.* | 258 | 5 | 0 | | | | | |
| Savin et al . | 10.1016/j.scitotenv. | 2021 | Germany | water samples (slaughte | *Klebsiella spp.* | 82 | 8 | | | | | | |

| | | | | | | | | | | | | | |
|---|---|---|---|---|---|---|---|---|---|---|---|---|---|
| | 2021.150000 | | | rhouse) + area of slaughter houses | | | | | | | | | |
| Savin et. al | 10.3390/antibiotics11040435 | 2022 | Germany | slaughter house wasterwater (poultry and pig; indistinguishable) | *Klebsiella spp.* | | 58 | | | 0.00% | | | |
| Sgariglia et al. | 10.12834/VetIt.1617.8701.1 | 2019 | Italy | liver + intestinal swab | *E. coli* | | | | | 0.00% | | | |
| Sghaier et. al | 10.1016/j.jgar.2019.01.002 | 2019 | Tunisia | fecal samples | *E. coli* | 60 | 75 | 0 | 0.00% | 0.00% | | | |
| Varga et. al | 10.1186/s12917-019-2187-z | 2019 | Canada | ceacal samples | *E. coli* | | | 0 | | | | | |
| Vogt et. al | 10.1089/mdr.2013.0210 | 2014 | Switzerland | meat samples | *E. coli* | 75 | | 0 | 0.00% | | | | |
| Zarfel et. al | 10.3390/ijerph111212582 | 2014 | Austria | meat samples | *E. coli* | 50 | 358 | 0 | 0.00% | | | | |

***Abbreviations: n = number; Pheno-R = phenotypic resistance; Geno-R = genotypic resistance; S = per samples; I = per isolates; Year = year of publication; Country = country where samples were collected***
