# Supplementary material for: Antimicrobial Resistance in Selected Enterobacteriaceae from Broilers and Their Environment: ESBL, AmpC, Carbapenemases, Colistin, and Fluoroquinolone Resistance—A Systematic Review and Meta-Analysis
Source: Antibiotics (Basel). 2025 Dec 15;14(12):1268. doi: 10.3390/antibiotics14121268 (PMC12865486; doi:10.3390/antibiotics14121268)
Supplement: Supplementary file 1 [file antibiotics-14-01268-s001.zip › antibiotics-3970136-supplementary/Supplementary_Table_S4_Colistin.pdf]

Table S4. Studies of colistin resistance (48)

Author	DOI	Year	Country	Sample type	Bacteria	n (samples)	n (isolates)	Pheno-R (n)	Pheno-R (S)	Pheno-R (I)	Geno-R (n)	Geno-R (S)	Geno-R (I)
Perrin-Guyomard et. al	http://dx.doi.org/10.2807/1560-7917.ES.2016.21.6.30135	2016	France	caecal samples	*E. coli*		621	7		1.13%	7		1.13%
El Garch et. al	10.1016/j.vetmic.2017.11.014	2018	Hungary, Germany, Spain, Netherlands, France, UK	caecal samples	*E. coli*	3581	3581	53	1.48%	1.48%	44	1.23%	1.23%
Iancu et. Al (no findings presented)		2018	Romania	carcasses (supermarkt)	*E. coli*	30	30	11		36.67%			
Zając et. Al (no findings presented)	10.3389/fmicb.2019.01753	2019	Poland	fecal samples	*E. coli*		1352	only moleculare detection			11		0.8%
Mohamed et. al (no findings presented)	10.1155/2014/195189	2014	Egypt	organ samples + cloacal swabs	*E. coli*	303	25	14	4.62%	investigation of several samples types			
Hanon et al.	10.1016/j.prevetmed.2015.09.001	2015	Belgium	caecal samples	*E. coli*	1132	1132	no persisting high prevalence <40%, ca. 2-3%					
Clemente et. al	10.1016/j.ijfoodmicro.2019.02.006	2019	Portugal	caecal samples	*E. coli*		202	6		2.97%	4	1.98%	66.67%
Agabou et al.	10.1007/s10096-015-2534-3	2015	Algeria	fecal samples	*E. coli*	70		0	0.00%				

Majewski et. al	10.2478/jvetres-2020-0060	2020	Poland	cloacal samples	*E. coli*	158	158	25	15.82%	15.82%	25	15.82%	
Kluytmans–van den Bergh et. al	10.2807/1560-7917.ES.2016.21.9.30149	2016	Netherlands	meat samples	*E. coli, Enterobacter spp.*		196	2		1.07%	3		1.60%
Myrenås et al.	10.1016/j.vetmic.2017.11.015	2017	Norway, Sweden, Iceland	ceacal samples + meat samples (nicht unterscheidbar)	*E. coli*		319 (nicht unterscheidbar zwischen caecal und meat samples)	1		0.31%			
Manageiro et. al	10.1016/j.ijfoodmicro.2017.10.007	2017	Portugal	caecal samples	*E. coli*	680	202	6	3.00%	3.00%	0	0.00%	0.00%
Majewski et. al	10.26444/aaem/120927	2020	Poland	organ samples	*E. coli, Klebsiella spp.*		4575	690		15.36%			
Alba et. al	10.3389/fmicb.2018.01217	2018	Italy	caecal samples	*E. coli*	300	300	9	3.00%	5.29%	8	2.67%	4.71%
Chaalal et. al	10.1089/mdr.2019.0419	2019	Algeria	meat samples	*Klebsiella spp.*	181							
Much et. al	10.1016/j.prevetmed.2019.104755	2019	Austria	caecal samples	*E. coli*	1031	962	0	0.00%				
Ribeiro et. al	10.1111/1462-2920.15689	2021	Portugal	chicken meat	*E. coli, Klebsiella spp.*								
Pesciaroli et. al	10.1016/j.ijfoodmicro.2019.108391	2019	Italy	caecal samples	*E. coli*	855	854	15	1.76%	1.76%	11	1.29%	73.33 %
Messaili et al.	10.12834/VetIt.799.3865.2	2019	Algeria	intestine	*E. coli*	100	100	0	0.00%	0.00%			

Hassen et. al	10.1016/j.ijfoodmicro.2019.108478	2019	Tunisia	fecal samples + meat samples	*E. coli*	333	333	54	16.22%	57,4% (53,1% faeces; 66,7% meat)	52	15.62%	15.62 %
Saidani et. al	10.1089/mdr.2019.0138	2019	Tunisia	cloacal samples	*E. coli, Klebsiella spp.*	258		5	1.94%	10.00%	5	1.94%	10.00 %
Mesa-Varona et al.	10.1371/journal.pone.0243772	2020	Germany	caecal samples	*E. coli*		592	25		4,2% (25), non-clinical 1% und clinical 5,7%			
Savin et. al	10.1128/AEM.02748-19	2020	Germany	wastewater (slaughter house)	*E. coli, Enterobacter spp., Klebsiella spp., Citrobacter spp.*	82		18	21.95%	9.68%	13	6.99%	72.22 %
Randall et. al	10.1111/jam.14687	2020	UK	meat samples	*E. coli*	622	283	0	0.00%	0.00%			
Dhaouadi et. al	10.1016/j.jgar.2020.03.017	2020	Tunisia	organ samples	*E. coli*	100	50	12	12.00%	24.00%	7	7.0%	58.33 %
Enany et. al (no findings presented)	10.1186/s13568-019-0920-4	2019	Egypt	environmental samples + organ samples of diseased animals	*E. coli*	368	152	0	0.00%	decision: excluded from prevalence calculation; investigation of several samples types			
Ibrahim et. al (no findings presented)	10.14202/vetworld.2019.141-145	2019	Egypt	organ samples	*E. coli*	1500	510	375	26.47%	26.47%			
Homeier-Bachmann et. al	10.3390/antibiotics10050568	2021	Germany	wastewater (slaughter house)	*E. coli, Enterobacter spp.,*		26	8		30.77%			

					Klebsiella spp.,								
Savin et al .	10.1016/j.scitotenv.2021.150000	2021	Germany	water samples (slaughter house) + area of slaughterhouses	*Klebsiella spp.*	82	71						
Facciolà et. al (no findings presented)	10.3390/ijerph18189611	2021	Italy	wastewater (slaughter house)	*E. coli, Citrobacter spp.*		5	1					
Musa et al.	10.3390/antibiotics10111321	2021	Italy	cloacal samples, ceacal samples, skin samples	*E. coli*		58	0					
Badr et. al	10.3390/vetsci9060282	2022	Egypt	organ samples	*E. coli*	120	56	23	41.07%	41.07%	56	41.07%	100.00 %
Chaalal et. al (no findings presented)	10.1089/mdr.2020.0109	2021	Algeria	meat samples	*E. coli, Klebsiella spp.*	181		17	9.39%				
Elmonir et. al	10.3390/biology10050373	2021	Egypt	organ samples + litter + water + food samples	*Klebsiella spp.*	160	19				2		10.53 %
Sadek et. al (no findings presented)	10.3390/microorganisms9010195	2021	Egypt	neck skin	*E. coli*	345		19	5.51%		20	5.80%	

Abbreviations: n = number; Pheno-R = phenotypic resistance; Geno-R = genotypic resistance; S = per samples; I = per isolates; Year = year of publication; Country = country where samples were collected
