# Supplementary material for: Antimicrobial Resistance in Selected Enterobacteriaceae from Broilers and Their Environment: ESBL, AmpC, Carbapenemases, Colistin, and Fluoroquinolone Resistance—A Systematic Review and Meta-Analysis
Source: Antibiotics (Basel). 2025 Dec 15;14(12):1268. doi: 10.3390/antibiotics14121268 (PMC12865486; doi:10.3390/antibiotics14121268)
Supplement: Supplementary file 1 [file antibiotics-14-01268-s001.zip › antibiotics-3970136-supplementary/Supplementary_Table_S6_Figure3_GenotypicR_per isolates.pdf]

Table S6. Genotypic resistance per isolates by resistance pattern (67)

**Author**	**Title**	**Country**	**Geno-R ESBL (I) CTX-M, SHV, TEM**	**Geno-R AmpC (I) CMY-2**	**Geno-R Carbapenem (I) OXA, KPC, VIM, NDM**	**Geno-R Colistin (I) mcr-1**	**Geno-R PMQR (I) qnr, qep, aac(6')-Ib**
Abbassi et. al	Genetic Background of Antimicrobial Resistance in Multiantimicrobial-Resistant Escherichia coli Isolates from Feces of Healthy Broiler Chickens in Tunisia	Tunisia	1.20%				
Abdallah et. al	Extended-Spectrum β-Lactamases and/or Carbapenemases-Producing Enterobacteriaceae Isolated from Retail Chicken Meat in Zagazig, Egypt	Egypt	65.09%		11.32%		
Awad et. al	Genetic elements associated with antimicrobial resistance among avian pathogenic Escherichia coli	Egypt	30.17%				33.30%
Badr et. al	Multidrug-Resistant and Genetic Characterization of Extended-Spectrum Beta-Lactamase-Producing E. coli Recovered from Chickens and Humans in Egypt	Egypt	75.00%		17.86%		
Badr et. al	Phenotypic and Genotypic Screening of Colistin Resistance Associated with Emerging Pathogenic	Egypt				100.00%	

	Escherichia coli Isolated from Poultry						
Ben Sallem et. al	Prevalence and Characterization of Extended-Spectrum Beta-Lactamase (ESBL)– and CMY-2–Producing Escherichia coli Isolates from Healthy Food-Producing Animals in Tunisia	Tunisia		9.09%			
Benameur et. al	Virulence, Antimicrobial Resistance and Biofilm Production of Escherichia coli Isolates from Healthy Broiler Chickens in Western Algeria	Algeria	55.56%				
Hassen et. al	High prevalence of mcr-1 encoding colistin resistance and first identification of blaCTXM55 in ESBL/CMY-2-producing Escherichia coli isolated from chicken faeces and retail meat in Tunisia	Tunisia	27.03%	1.20%		15.62%	
Baron et. al	Impact of Third-Generation-Cephalosporin Administration in Hatcheries on Fecal Escherichia coli Antimicrobial Resistance in Broilers and Layers	France	19.15%	5.60%			
Belmar Campos et. al	Prevalence and genotypes of extended spectrum beta-lactamases in Enterobacteriaceae isolated from human stool and chicken meat in Hamburg, Germany	Germany	100.00%				

Casella et. al	High prevalence of ESBLs in retail chicken meat despite reduced use of antimicrobials in chicken production, France	France	100.00%	3.90%				
Chalmers et. al	Extended-spectrum cephalosporin resistance in Escherichia coli from broiler chickens raised with or without antibiotics in Ontario, Canada	Canada	18.35%	82.57%				
Clemente et. al	Prevalence and Characterization of ESBL/AmpC Producing Escherichia coli from Fresh Meat in Portugal	Portugal	100.00%	1.67%			5.00%	38.33%
Cohen Stuart et. al	Comparison of ESBL contamination in organic and conventional retail chicken meat	Netherlands	49.69%					
Costa et. al	Prevalence of extended-spectrum beta-lactamase-producing Escherichia coli isolates in faecal samples of broilers	Portugal	91.18%					
Ghodousi et. al	Extended-Spectrum ß-Lactamase, AmpC-Producing, and Fluoroquinolone-Resistant Escherichia coli in Retail Broiler Chicken Meat, Italy	Italy	98.51%	11.19%				81.34%
Dhaouadi et. al	Co-occurrence of mcr-1 mediated colistin resistance and β-lactamase-encoding genes in multidrug-resistant Escherichia coli from broiler chickens with colibacillosis in Tunisia	Tunisia	83.33%				58.30%	

Diarrassouba et. al	Antibiotic Resistance and Virulence Genes in Commensal Escherichia coli and Salmonella Isolates from Commercial Broiler Chicken Farms†	Canada		80.00%			
Dierikx et. al	Increased detection of extended spectrum beta-lactamase producing Salmonella enterica and Escherichia coli isolates from poultry	Netherlands	95.45%	22.73%			
Elmonir et. al	Emergence of Colistin and Carbapenem Resistance in Extended-Spectrum β-Lactamase Producing Klebsiella pneumoniae Isolated from Chickens and Humans in Egypt	Egypt	68.42%		21.05%	10.53%	
El-Shazly et. al	Expanded spectrum β–lactamase producing Escherichia coli isolated from chickens with colibacillosis in Egypt	Egypt	18.00%	12.00%			
Forward et. al	Recovery of cephalosporin-resistant Escherichia coli and Salmonella from pork, beef and chicken marketed in Nova Scotia	Canada		90.70%			
Gregova et. al	Antibiotic resistance of Escherichia coli isolated from a poultry slaughterhouse	Slovakia		33.33%			
Hadžić-Hasanović et. al	Phenotypic and genotypic detection of ESBL-producing E. coli isolates from chicken skin in Bosnia and Herzegovina	Bosnia and Herzegovina	82.76%				
Friese et. al	Faecal occurrence and emissions of livestock-	Germany	23.08%	38.46%			

	associated methicillin-resistant Staphylococcus aureus (laMRSA) and ESBL/AmpC-producing E. coli from animal farms in Germany						
Kaesbohrer et. al	Diversity in prevalence and characteristics of ESBL/pAmpC producing E. coli in food in Germany	Germany	71.88%	26.04%			
Leverstein-van Hall et. al	Dutch patients, retail chicken meat and poultry share the same ESBL genes, plasmids and strains	Netherlands	100.00%				
Blanc et. al	ESBL- and plasmidic class C b-lactamase-producing E. coli strains isolated from poultry, pig and rabbit farms	Spain		6.77%			
Majewski et. al	Colistin resistance of non-pathogenic strains of Escherichia coli occurring as natural intestinal flora in broiler chickens treated and not treated with colistin sulphate	Poland				15.82%	
Machado et. al	Antibiotic resistance integrons and extended-spectrum b-lactamases among Enterobacteriaceae isolates recovered from chickens and swine in Portugal	Portugal	15.84%				
Messaili et al.	Virulence gene profiles, antimicrobial resistance and phylogenetic groups of fecal Escherichia coli strains isolated from	Algeria	70.00%				13.00%

	broiler chickens in Algeria						
Michael et. al	Extended-spectrum b-lactamase (ESBL)-producing Escherichia coli isolates collected from diseased food-producing animals in the GERM-Vet monitoring program 2008–2014	Germany	0.50%				
Moawad et. al	Occurrence of Salmonella enterica and Escherichia coli in raw chicken and beef meat in northern Egypt and dissemination of their antibiotic resistance markers	Egypt	66.67%		20.00%		33.33%
Moawad et al. (Antimicrobial)	Antimicrobial resistance in Enterobacteriaceae from healthy broilers in Egypt: emergence of colistin-resistant and extended-spectrum β-lactamase-producing Escherichia coli	Egypt	20.00%	3.08%		7.94%	9.23%
Päivärinta et. al	Low Occurrence of Extended-Spectrum b-lactamase-Producing Escherichia coli in Finnish Food-Producing Animals	Finland	31.71%	53.66%			
Perrin-Guyomard et. al	Prevalence of mcr-1 in commensal Escherichia coli from French livestock, 2007 to 2014	France				7.94%	
Poudel et. al	Multidrug-Resistant Escherichia coli, Klebsiella pneumoniae and Staphylococcus spp. in Houseflies and Blowflies from Farms and Their	USA	0.00%	0.00%			

	Environmental Settings						
Ramadan et. al	Phenotypic and genetic characterization of β-lactam resistance in Klebsiella from retail chicken meat in Mansoura, Egypt	Egypt	84.60%				
Ramadan et. al	Antimicrobial Resistance, Genetic Diversity and Multilocus Sequence Typing of Escherichia coli from Humans, Retail Chicken and Ground Beef in Egypt	Egypt			0.00%		
Ramadan et. al	Contribution of Healthy Chickens to Antimicrobial-Resistant Escherichia coli Associated with Human Extraintestinal Infections in Egypt	Egypt	32.00%	34.00%			
Randall et. al	A decline in the occurrence of extended-spectrum b-lactamase-producing Escherichia coli in retail chicken meat in the UK between 2013 and 2018	UK	84.56%				
Sadek et. al	Genomic Features of MCR-1 and Extended-Spectrum β-Lactamase-Producing Enterobacterales from Retail Raw Chicken in Egypt	Egypt				100.00%	
Savin et. al	Antibiotic-resistant bacteria, antibiotic resistance genes, and antibiotic residues in wastewater from a poultry slaughterhouse after	Germany	42.27%				

	conventional and advanced treatments						
Sheikh et. al	Antimicrobial Resistance and Resistance Genes in Escherichia coli Isolated from Retail Meat Purchased in Alberta, Canada	Canada	75.13%	26.94%			
Solà-Ginés et. al	Houseflies (Musca domestica) as Vectors for Extended-Spectrum-Lactamase-Producing Escherichia coli on Spanish Broiler Farms	Spain	100.00%	0.00%			
Zając et. al	Occurrence and Characterization of mcr-1-Positive Escherichia coli Isolated From Food-Producing Animals in Poland, 2011–2016	Poland				0.81%	
Huijbers et. al	Extended-spectrum and AmpC b-lactamase-producing Escherichia coli in broilers and people living and/or working on broiler farms: prevalence, risk factors and molecular characteristics	Netherlands	61.36%	38.64%			
Alba et. al	Molecular Epidemiology of mcr-Encoded Colistin Resistance in Enterobacteriaceae From Food-Producing Animals in Italy Revealed Through the EU Harmonized Antimicrobial Resistance Monitoring	Italy				4.71%	
Belmahdi et al .	Molecular characterisation of extended-spectrum b-	Algeria	80.00%	20.00%			

	lactamase- and plasmid AmpC-producing Escherichia coli strains isolated from broilers in Bejaıa, Algeria						
Clemente et. al	Revealing mcr-1-positive ESBL-producing Escherichia coli strains among Enterobacteriaceae from food-producing animals (bovine, swine and poultry) and meat (bovine and swine), Portugal, 2010–2015	Portugal				66.67%	
El Garch et. al	mcr-1-like detection in commensal Escherichia coli and Salmonella spp. from food-producing animals at slaughter in Europe	Hungary, Germany, Spain, Netherlands, France, UK				1.23%	
Hamza et. al	Carbapenemase-producing Klebsiella pneumoniae in broiler poultry farming in Egypt	Egypt			42.86%		
García-Béjar et. al	High Prevalence of Antibiotic-Resistant Escherichia coli Isolates from Retail Poultry Products in Spain	Spain	65.00%				
Kluytmans–van den Bergh et. al	Presence of mcr-1-positive Enterobacteriaceae in retail chicken meat but not in humans in the Netherlands since 2009	Netherlands				1.60%	
Kmet et al.	High Level of Quinolone Resistance in Escherichia coli from Healthy Chicken Broilers	Slovakia	0.00%				0.32%

Kocúreková et al.	Antimicrobial Susceptibility and Detection of Virulence-Associated Genes in Escherichia coli Strains Isolated from Commercial Broilers	Slovakia	1.74%				
Liebana et. al	Characterization of b-Lactamases Responsible for Resistance to Extended-Spectrum Cephalosporins in Escherichia coli and Salmonella enterica Strains from Food-Producing Animals in the United Kingdom	UK	50.00%				
Manageiro et. al	New insights into resistance to colistin and third-generation cephalosporins of Escherichia coli in poultry, Portugal: Novel blaCTXM166 and blaESAC genes	Portugal	5.45%	5.45%		0.00%	
Merchant et. al	Characterization of antibiotic-resistant and potentially pathogenic Escherichia coli from soil fertilized with litter of broiler chickens fed antimicrobial-supplemented diets	Canada	14.92%	32.20%			
Niero et al.	High diversity of genes and plasmids encoding resistance to third-generation cephalosporins and quinolones in clinical Escherichia coli from commercial poultry flocks in Italy	Italy	9.18%	2.04%			4.08%
Päivärinta et. al	Whole genome sequence analysis of	Finland		18.84%		0.00%	

	antimicrobial resistance genes, multilocus sequence types and plasmid sequences in ESBL/AmpC Escherichia coli isolated from broiler caecum and meat						
Pesciaroli et. al	Antibiotic-resistant commensal Escherichia coli are less frequently isolated from poultry raised using non-conventional management systems than from conventional broiler	Italy	79.31%			73.33%	
Savin et al .	Slaughterhouse wastewater as a reservoir for extended-spectrum β-lactamase (ESBL)-producing, and colistin-resistant Klebsiella spp. and their impact in a “One Health” perspective	Germany	88.73%				4.23%
Chenouf et. al	Extended Spectrum b-Lactamase-Producing Escherichia coli and Klebsiella pneumoniae from Broiler Liver in the Center of Algeria, with Detection of CTX-M-55 and B2/ST131-CTX-M-15 in Escherichia coli	Algeria	16.67%				
Maciuca et. al	High Prevalence of Escherichia coil-Producing CTX-M-15 Extended-Spectrum Beta-Lactamases in Poultry and Human Clinical Isolates in Romania	Romania	100.00%	53.33%			

Vogt et. al	Occurrence and Genetic Characteristics of Third-Generation Cephalosporin-Resistant Escherichia oil/ in Swiss Retail Meat	Switzerland	80.88%	23.64%			
Literak et. al	Broilers as a Source of Quinolone-Resistant and Extraintestinal Pathogenic Escher-ch-a col- in the Czech Republic	Czech Republic					4.30%

Abbreviations: ESBL = Extended-spectrum Beta-lactamase (CTX-M, SHV, TEM); AmpC = AmpC Beta-lactamase (CMY_2); OXA, KPC, VIM, NDM = Carbapenemase genes; mcr-1 = Colistin resistance gene; PMQR = Plasmid-mediated quinolone resistance genes (qnr, qep, aac(6')-Ib)
